# Integrated optimization of scheduling for unmanned follow-me cars on airport surface

**DOI:** 10.1038/s41598-024-58918-7

**Published:** 2024-04-12

**Authors:** Dezhou Yuan, Xinping Zhu, Yajun Zou, Qing Zhao

**Affiliations:** College of Air Traffic Management, Civil Aviation Flight University of China, Deyang, 618307 China

**Keywords:** Applied mathematics, Computational science, Aerospace engineering, Civil engineering, Energy infrastructure

## Abstract

To promote the application of automated vehicles in large airports, in this study, we present an integrated optimization method for scheduling Unmanned follow-me cars. The scheduling process is divided into three phases: Dispatch, Guidance, and Recycle. For the Dispatch phase, we establish a vehicle assignment model, to allocate the vehicle resource equitably. For the Guidance phase, we offer an quantitative way, to measure the spacing between Unmanned follow-me car and aircraft. To optimize the efficiency of airport operation in the three phases and ensure safety, the collaborative planning model, and the conflict prediction model are established. An improved grey wolf optimization algorithm is adopted to enhance the convergence speed and generalization performance. A case study at Ezhou Huahu Airport in China demonstrates the effectiveness of the methods. The results show that the model of collaborative planning can make the balance of path selection, Unmanned follow-me car’s working time, and departure sequence. The convergence speed of the improved algorithm has been increased by 18.75%. The inequity index of vehicle assignment is only 0.015731, and the spatiotemporal distribution of conflicts is influenced by the airport’s surface layout.

## Introduction

With the recovery of civil aviation after the epidemic of COVID-19, the introduction of unmanned vehicles at airports is considered as an innovative measure to improve airport operational efficiency and reduce costs. These vehicles, equipped with advanced sensors, controllers, enable autonomous driving functions with complex environment perception, intelligent decision-making, and collaborative intelligence. They have become the new generation of airport equipment for operations and support^1^. However, airport operation is a critical issue about safety, and it requires full consideration. Now that a single unmanned vehicle, has the capability for automatic control^2^, the overall risk control of the aviation transportation system in the hermetic environment of airport surface has become the focus. How to conduct scheduling of the fleet scientifically is a major problem for airports.

To balance the security and efficiency of the fleet, scholars have conducted research on the scheduling problem of unmanned vehicles in the airport scene. Basically, the scheduling process involves path planning and vehicle assignment^3^. For path planning, an optimized combination model is introduced. Because this model needs to discuss the situation in different velocities of the vehicles, it was solved with the heuristic, such as hill climbing particle swarm optimization (HC-PSO) algorithm^4^. For the other, the utilization ratio is considered as a kind of index, to evaluate the equity of vehicle assignment. A dynamic programming model with various charging strategies meets the needs of allocation for vehicle resource^5^. Generally, the scheduling problem is a classic vehicle routing problem with time windows (VRPTW). Different models and algorithms have been explored to address this challenge, including linear programming (LP), combinatorial optimization, graph theory, and network analysis^6^. Common algorithms include exhaustive methods, C-W savings algorithms, column generation algorithms, heuristic search, genetic algorithms (GA), and particle swarm optimization (PSO) algorithms. However, research on unmanned vehicle scheduling on airport surface mostly focused on electric tractors, which are subject to traction speed limitations^7^ and do not meet the requirements of large-scale airports. There is a lack of research on the scheduling process of unmanned follow-me cars (UFMCs).

Regarding VRPTW on airport surface, integer linear programming (ILP) algorithms is utilized to determine the optimal allocation of ground support equipment (GSE) based on cost and time constraints^8^. The sorting of flight support operations for airport service vehicles is improved by GA, to optimize the structure and quantity of service vehicles. The objective is to enhance the efficiency of service vehicle utilization^9^. An energy consumption model for GSE is developed by the adaptive large neighborhood search (ALNS) algorithm, and provides scheduling solutions for scenarios involving both fuel-powered and electric vehicles, addressing the challenge of minimizing energy consumption^10^. Dispatching issue for electric GSE in the apron is addressed by hybrid neural networks (HNN), contributing to the overall improvement in efficiency^11^. An intelligent dispatching model for support vehicles is solved by GA, with a focus on optimizing the allocation of GSE and efficiency^12^. A theoretical system based on ILP is developed to solve the assignment problem of airport operational units, providing an effective framework for optimizing the allocation of operational units within the airport^13^. Collaborative decision-making for parking allocation and shuttle scheduling is successfully implemented through column generation (CG) algorithm, aiming to improve the efficiency of parking allocation and shuttle scheduling processes^14^. An objective function that minimizes operating costs and flight delay costs and optimizes GSE scheduling is devised with the help of ILP, to reduce operational costs and minimize flight delays through GSE scheduling^15^. Heuristic search methods are employed in a real-time scheduling decision system to efficiently assign GSE^16^.

Regarding aircraft path planning on airport surfaces, an ILP mathematical model for conflict-free aircraft trajectories in various traffic density scenarios is established with a rolling time domain calculation strategy^17^. A mixed ILP (MILP) model for gate allocation is developed by combining operation modes of multiple runway airports, aiming to optimize gate allocation^18^. Heuristic methods are implemented to search for the optimal speed profile for ground taxiing process, providing valuable references for aerodrome control units^19^. Optimal taxiing speed profiles were generated with an exhaustive method based on required time of arrival (RTA)^20^. Surface routing planning problems are also studied through multi-agent simulation^21^.

In conclusion, there are various optimization techniques employed in scheduling UFMCs on airport surfaces, including ILP, GA, ALNS, HNN, and heuristic search methods. Additionally, mathematical models such as ILP and MILP are utilized for path planning on airport surface. Some professional concepts such as RTA, energy management and flow of transportation management are introduced, that makes mathematical models meet the demands of business processes. However, further exploration is needed to develop more efficient and accurate optimization techniques for the scheduling of UFMCs on airport surface.

While previous research has provided insights into the issue of scheduling on airport surfaces, these studies have some limitations. Firstly, they mainly consider vehicles for small to medium-scale operations, typically involving up to 50 flights, which may not meet the demands of larger-scale vehicle scheduling. Secondly, although the vehicle assignment models may generate satisfactory solutions, they often fail to take uncertainties and flight support requirements into account. Finally, the evaluation of potential movement conflicts is not thoroughly addressed in these studies. The conflicts are either treated as disturbances or some mitigation suggestions are provided.

**Figure 1:**
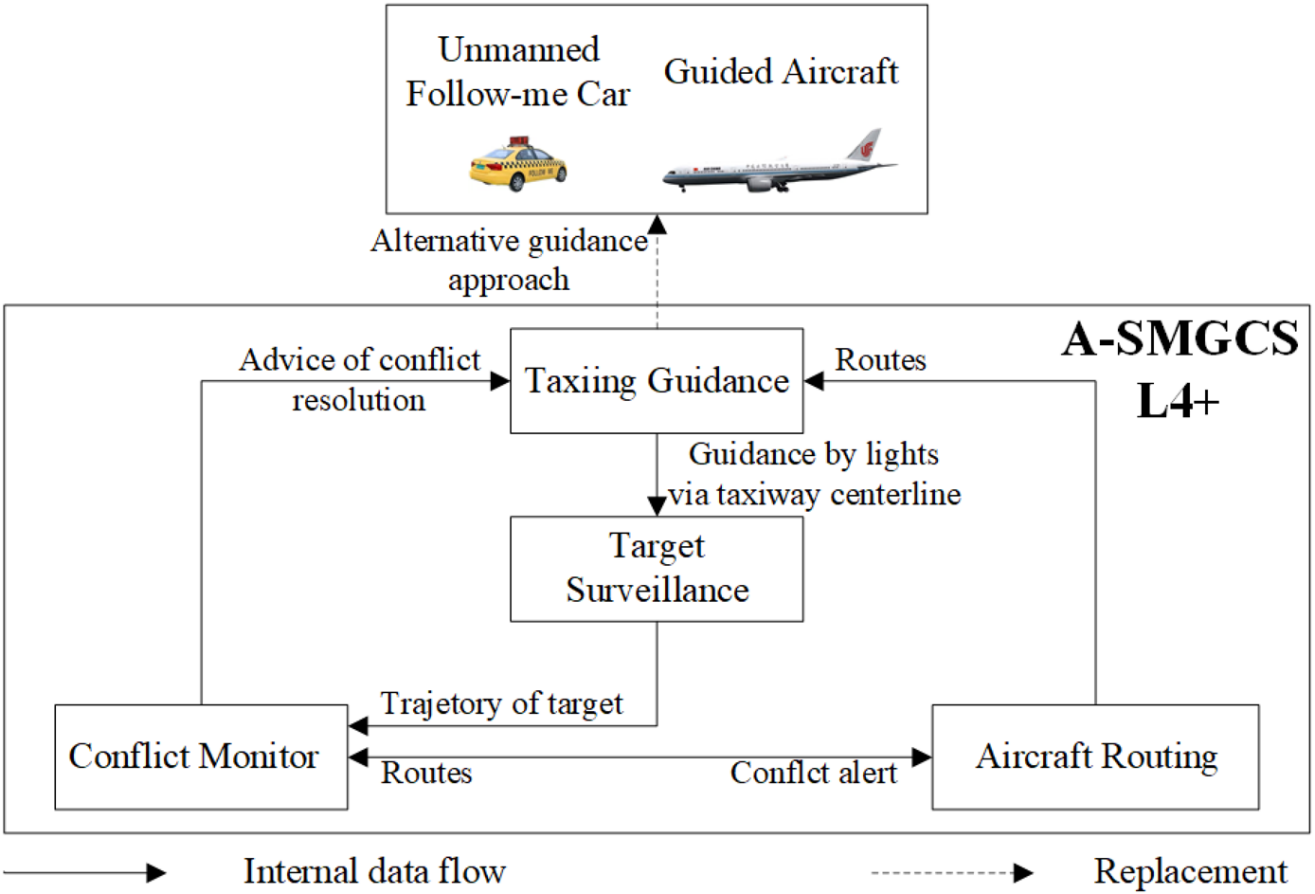
Working principle of the UFMC scheduling system.

On the other hand, in advanced surface movement guidance and control systems (A-SMGCS) Level 4 and above, the surface movement of aircraft relies on three functional modules: target surveillance, conflict alert, and routing. However, the conventional method of surface taxiing guidance with lights of centerline on the taxiway proves to be expensive in terms of maintenance. It is not suitable for large and busy airports. To address this issue, an alternative approach within a generalized A-SMGCS implementation framework is proposed, which utilizes UFMCs as a replacement for taxiing guidance. Figure 1 shows the basic working principle of this approach, where the UFMC scheduling system integrates with the existing functional modules. In this concept, the aircraft follows the UFMC during taxiing, requiring close attention from the pilot for guidance. The advantage of UFMCs over traditional manual follow-me cars lies in their ability to formulate efficient vehicle scheduling schemes, enabling prompt departures from the parking lot and seamless integration with guidance tasks. Furthermore, UFMCs overcome the drawback of slower towing speeds associated with electric tractors, ensuring the efficiency of airport surface operations. Therefore, this paper aims to study the integrated optimization of UFMC scheduling to address this guidance requirement.

## Problem description

**Figure 2:**
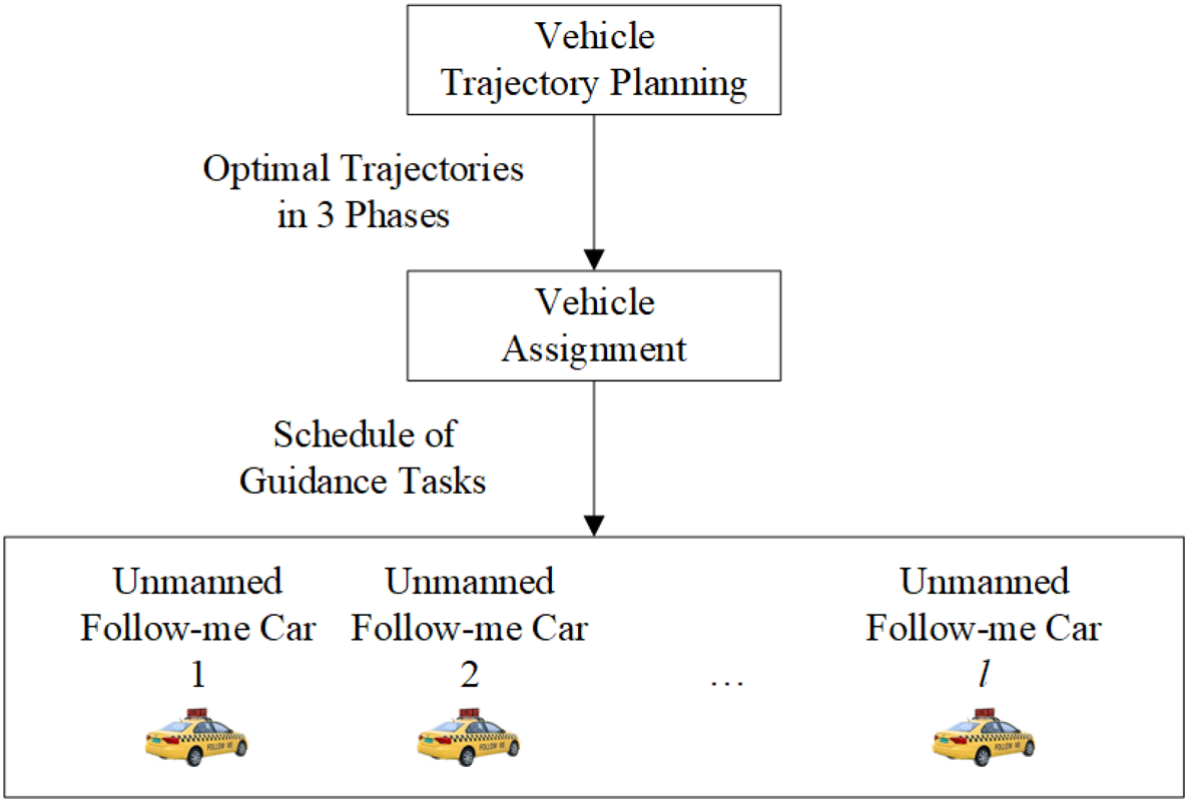
The integrated scheduling process for UFMCs.

The integrated scheduling process for UFMCs to facilitate aircraft taxiing guidance is illustrated in Fig. 2. This process comprises vehicle trajectory planning and vehicle assignment. Vehicle trajectory planning involves generating optimal trajectories for the UFMCs across three phases: dispatch from the parking lot to the start of guidance (Dispatch), implementation of the guidance process (Guidance), and return to the parking lot after completing the guidance (Recycle). These trajectories are generated based on specific safety and efficiency objectives. Each vehicle’s trajectory consists of a sets of position coordinates $\left( x,y \right)$ along the route, corresponding velocities $\left( v \right)$, and timestamps $\left( t \right)$. Vehicle assignment refers to the selection of a specific UFMC from a pool of candidates based on certain optimization objectives to perform ground taxiing guidance for the corresponding flight.

As can be observed, the precise planning of UFMCs in terms of starting, stopping, and speed profiles enables a higher level of refinement compared to manually operated follow-me cars. This allows vehicles to promptly reach the starting point of the guidance task and proactively plan to avoid potential conflicts during their operations. For the convenience of discussion, the UFMC and the aircraft being guided by it are considered as a “Guidance Unit (GU)” in the surface movement scenario.

**Figure 3:**
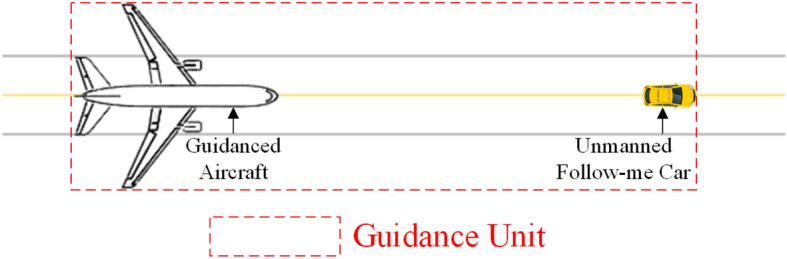
Schematic diagram of a GU.

From Fig. 3, during the Guidance phase, there exists a following relationship between the aircraft and the UFMC. The spacing between them affects the spatial occupancy of the GU. It also influences the scheduling decisions. To address this, a definition and a quantification method for the distance are provided:

### Definition 1

(*Guidance following spacing*) Within the GU, the spacing $\Delta L$ from the front of the aircraft, to the rear of the UFMC.

**Figure 4:**
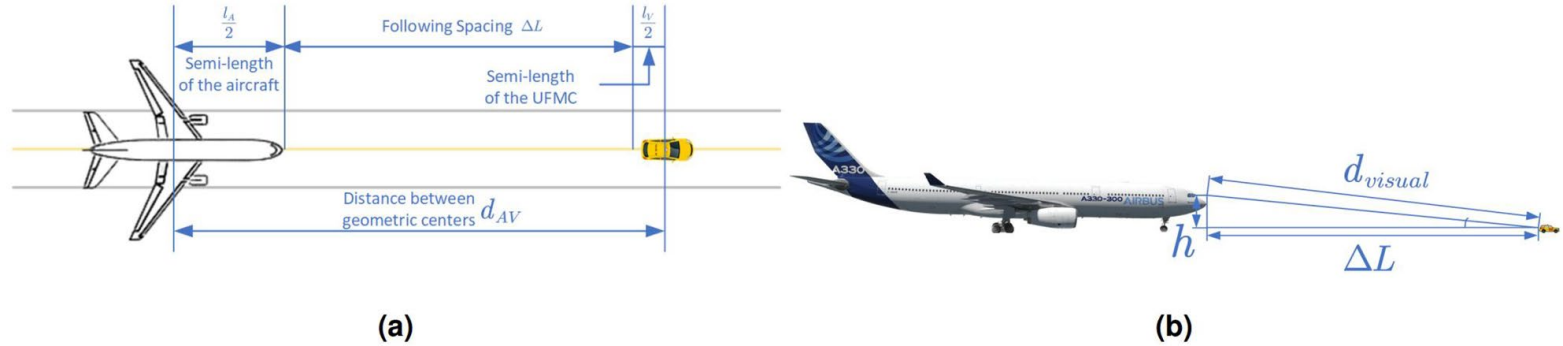
Measurement method for guidance following spacing. **(a)** Vertical view. **(b)** Lateral view.

As shown in Fig. 4a, the measurement of $\Delta L$ is similar to the concept of Following Spacing in transportation engineering^22^:

1 $$\begin{aligned} \Delta L=d_{AV}-\frac{l_A}{2}-\frac{l_V}{2} \end{aligned}$$

where $l_A$ and $l_V$ are the lengths of the aircraft and the UFMC, respectively. $d_{AV}$ represents the distance between their geometric centers.

During the following process, the visual influence to the pilot must be considered in order to comprehensively evaluate the safe guidance following spacing for each type of aircraft. As depicted in Fig. 4b, there exists the following relationship between the visual spacing $d_{visual}$ in the cockpit, and the guidance following spacing $\Delta L$ is calculated by the Pythagorean theorem.

## Model formulation

**Figure 5:**
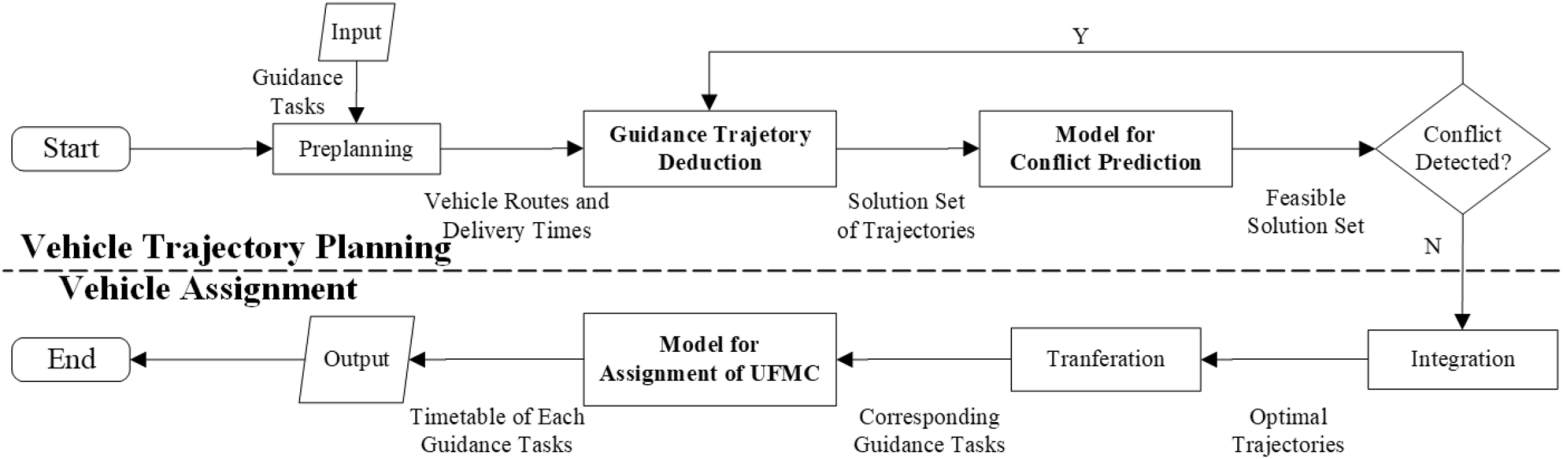
Framework of the scheduling model for UFMCs.

The scheduling model for UFMCs consists of two modules: vehicle trajectory planning and vehicle assignment. Figure  5 shows the details about this model:

- The vehicle trajectory planning module comprises the method for guidance trajectory deduction and a conflict prediction model. The former is responsible for rapidly deducting and generating a solution set of trajectories based on pre-planned vehicle routes and delivery times, with the input of guidance tasks. The latter is used to evaluate potential conflicts and determine the feasibility of the solution set. Finally, all feasible solution sets are integrated to generate an optimal solution set of trajectories.
- The vehicle assignment module transforms the optimal into corresponding guidance tasks, employing the UFMC assignment model to achieve the assignment function. Ultimately, the optimal scheduling scheme, i.e., the timetable of each guidance task, is obtained.

### Collaborative planning model for surface guidance trajectories

The set of flights to be guided denoted as $G=\left\{ g_1,g_2,\ldots ,g_s \right\}$, where *s* means the total number of guidance tasks, i.e., the total number of flights. The three phases of the guidance task are denoted as $P=\left\{ p_1,p_2,p_3 \right\}$, corresponding to the Dispatch phase, Guidance phase, and Recycle phase.

#### Objective function

To meet safety and efficiency requirements, the objective function is designed from the following perspectives:

- Minimizing the number of conflicts

The number of conflicts be denoted as $N_{conflict}$, and set the objective to minimize the number of conflicts:

2 $$\begin{aligned} \min N_{Conflict} \end{aligned}$$

- Minimizing the working time of UFMCs

Minimizing the working time of UFMCs means reasonable route planning, and reducing the ineffective waiting time of UFMCs at the gap between two phases as possible. Therefore, the objective is to minimize the working time of UFMCs:

3 $$\begin{aligned} \min \sum _{i=1}^s{\sum _{j=1}^3{t_{ij}}} \end{aligned}$$

Here, *i* represents the *i*-th guidance task in the timetable, *j* represents the phase at which the UFMC is located, and *s* is the total number of flights in the timetable. The working time of UFMCs is the sum of the working times in each phase of the guidance tasks, and it is measured in seconds. Therefore, it could reach the magnitude of 100,000. Meanwhile, the number of conflicts is up to 1000 extremely.

To achieve the comprehensive optimization of the two objectives, the range of working time of UFMCs is normalized to [0,1] as follows:

4 $$\begin{aligned} {\left\{ \begin{array}{ll} T=\sum \limits _{i=1}^s{\sum \limits _{j=1}^3{t_{ij}}}\\ T'=1-\frac{1}{T}\\ \end{array}\right. } \end{aligned}$$

Here, the working time of UFMCs is transformed into $T'$, to remove the influence of dimension while ensuring the monotonicity of the indicator.

Therefore, the objective function for vehicle trajectory planning is:

5 $$\begin{aligned} \min Z\,\,=\theta _1T'+\theta _2N_{conflict} \end{aligned}$$

While setting $\theta _1=1$, the integral part of the objective function value *Z* maps the number of conflicts on the field, and the decimal part maps the total working time of UFMCs on airport surface. The value of $\theta _2$ is determined by the scale of the airport surface, ensuring that $\forall N_{conflict}\in {\mathbb {N}}$, $\theta _2N_{conflict}$ is one order of magnitude larger than $\theta _1T'$^23^, achieving simultaneous optimization of the two components.

#### Constraints

- Constraints of routing for UFMCs

For the three phases of vehicle guidance, the UFMC scheduling system has prestored one shortest route and $\left( k^*-1 \right)$ sub-optimal routes for each pair of origins and destinations. During the process of scheduling, the system selects one from the $k^*$ candidates as the route of UFMC, i.e.,

6 $$\begin{aligned} R_{ij}\leqslant k^*, \quad \forall i\in G,j\in P \end{aligned}$$

where $R_{ij}$ is an integer decision variable that represents the number of the route. The subscript *i* and *j* indicate that route selection is performed for each guidance task and each phase in the trajectory planning module.

- Constraints of time windows for UFMCs

The aerodrome control unit and the Airport Operation Center (AOC) adjust the delivery time windows of the UFMCs through collaborative decision-making throughout the flight process to achieve integrated optimization of scheduling of UFMCs and control of flights:

7 $$\begin{aligned} \gamma _{ij}\leqslant \delta _j,\quad \forall i\in G,j\in P \end{aligned}$$

where $\gamma _{ij}$ is the decision variable, corresponding to the delivery time adjustment for the *i*-th guidance task in phase *j*. $\delta _j$ represents the upper limit of adjustment for each phase.

Taking guiding flight *i* as an example, the delivery times for the three phases of the UFMC are shown in Table 1. ETA and ETD are obtained from the flight schedule. They represent the Estimated Time of Arrival and Estimated Time of Departure, respectively. LDR is the time from the arrival aircraft touching down, to reaching the starting point of the Guidance. LUP is the time from the departure aircraft waiting at the runway entrance to having clearance of lining up the runway, respectively. CRS represents the time taken for the UFMC to pass through the taxiway on the apron, while UT is the time for the UFMC to make a U-turn in front of an arrival aircraft.

**Table 1:** Delivery time of UFMC guiding flight *i* in three phases.

Flight Type	Phase	Regular delivery time (RDT)	Actual delivery time (ADT)
Arrival	1	ETA+LDR-$t_{i1}$-UT	RDT-$\gamma _{i1}$
Arrival	2	ETA+LDR	RDT-$\gamma _{i2}$
Arrival	3	ETA+LDR+$t_{i2}$+CRS	RDT-$\gamma _{i2}+\gamma _{i3}$
Departure	1	ETD-LUP-$t_{i2}$-$t_{i1}$-CRS	RDT-$\gamma _{i1}-\gamma _{i2}-\gamma _{i3}$
Departure	2	ETD-LUP-$t_{i2}$	RDT-$\gamma _{i2}-\gamma _{i3}$
Departure	3	ETD-LUP	RDT-$\gamma _{i3}$

- Constraints of domain

To ensure the meaningfulness of each variable, it is necessary to define their domain, including the number of conflicts as a natural number, the route number of the UFMC as a positive integer, and the non-negativity of the UFMC working time and delivery time adjustment:

8 $$\begin{aligned}{} & {} N_{Conflict}\in {\mathbb {N}}\end{aligned}$$

9 $$\begin{aligned}{} & {} k,R_{ij}\in {\mathbb {N}} ^+, \quad \forall i\in G,j\in P\end{aligned}$$

10 $$\begin{aligned}{} & {} t_{ij},\gamma _{ij},\delta _j\geqslant 0,\quad \forall i\in G,j\in P \end{aligned}$$

#### Trajectory deduction

The purpose of guidance trajectory deduction is to deduce the trajectory of an UFMC or GU, for each guidance task at different phases based on a predetermined route and the delivery time. For UFMCs, precise control to parameters can be achieved based on the setting of guidance speeds in different zones, vehicle performance parameters, as well as road conditions, enabling accurate trajectory deduction to support the prediction of potential conflict in trajectories.

##### Definition 2

*Trajectory of UFMC* The coordinates the UFMC’s geometric center passes through, along with corresponding timestamps and velocities.

##### Definition 3

*Trajectory of GU* The coordinates the GU’s geometric center passes through, along with corresponding timestamps and velocities.

Since the preplanning process has generated several candidate routes, in order to accurately match the UFMC with the guidance requirements, further algorithm design is required, to generate high-precision velocity profiles and achieve synchronization between the actual delivery time and the start of the timestamp.

The input to the algorithm of Speed Profile Generation for UFMC is a set of nodes formed by a single candidate route. Then, correct the speed limit and determine the safety throttle/braking distance for each road segment, by basic kinematic equations. Finally, generate the final velocity profile:

11 $$\begin{aligned}{} & {} {\left\{ \begin{array}{ll} v_n=v_{n-1}\\ s_{n-1}^{n}=v_{n-1}\left( t_n-t_{n-1} \right) \\ \end{array}\right. }\end{aligned}$$

12 $$\begin{aligned}{} & {} {\left\{ \begin{array}{ll} v_n=v_{n}^{'}\\ t_n-t_{n-1}=\frac{s_{n-1}^{n}-s_{safe}}{v_n}+\frac{v_n-v_{n-1}}{a}\\ \end{array}\right. } \end{aligned}$$

13 $$\begin{aligned}{} & {} {\left\{ \begin{array}{ll} v_{n}^{2}-v_{n-1}^{2}=2as_{n-1}^{n}\\ s_{n-1}^{n}=\frac{v_n+v_{n-1}}{2}\left( t_n-t_{n-1} \right) \\ \end{array}\right. } \end{aligned}$$

The algorithm is showed as following:

**Algorithm 1:**
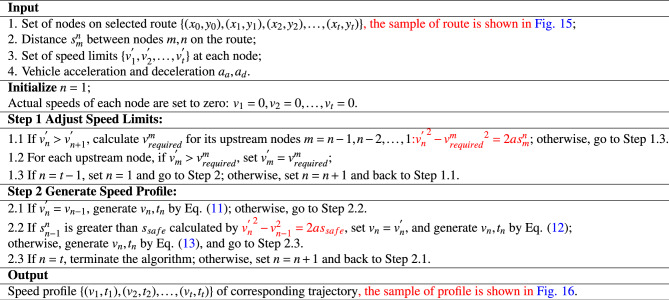
Speed Profile Generation for Unmanned Follow-me Car.

**Figure 6:**
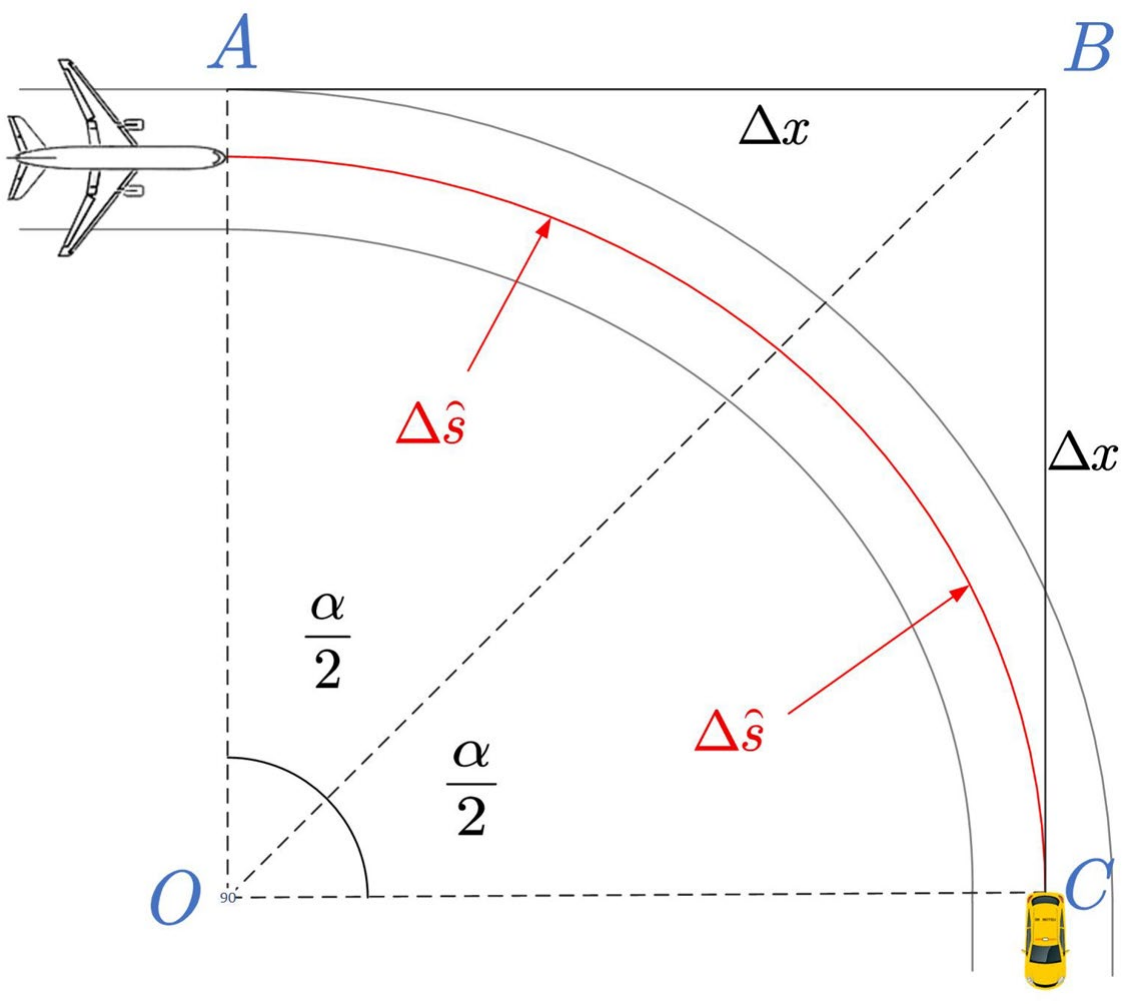
Smoothing for segments at turning areas.

The process for the guidance trajectory deduction is as follows:

- Input the current phase *j*, the number of parking stand where flight *i* is located, and the selected route $R_{ij}$. Generate the set of nodes$\left\{ \left( x_0,y_0 \right) ,\left( x_1,y_1 \right) ,\left( x_2,y_2 \right) ,\ldots ,\left( x_t,y_t \right) \right\}$corresponding to the guidance route;
- Input the delivery time adjustment $\gamma _{ij}$ and determine the delivery time, denoted as $t_0$, according to Table 1;
- Traverse the nodes on the trajectory, and determine the turning points via the dot product formula:
  14 $$\begin{aligned} \cos \alpha =\frac{\vec {AB}\cdot \vec {BC}}{\left| \vec {AB} \right| \left| \vec {BC} \right| } \end{aligned}$$
  Where point B is the point to be determined, and points A and C are the previous and next nodes, respectively. If $\alpha >60^{\circ }$, point B is identified as a turning point.
- Determine the speed limits $\left\{ v_{1}^{'},v_{2}^{'},\ldots ,v_{t}^{'} \right\}$ for each point based on actual operational conditions:
  15 $$\begin{aligned} v_{i}^{\prime } = v_{node} \quad (node \in {E_{Apron},E_{Taxiway},E_{Turn}}),\quad \forall i \in [1,t] \end{aligned}$$
  Where $v_{node}$ represents the speed limit at the next node, i.e., the current section’s speed limit. $E_{Apron}$, $E_{Taxiway}$, and $E_{Turn}$ represent the apron (including service roads), the taxiways in the maneuvering area, and the turning points, respectively;
- Call Algorithm 1 to generate the velocity profile $\left\{ \left( v_1,t_1 \right) ,\left( v_2,t_2 \right) ,\ldots ,\left( v_t,t_t \right) \right\}$:
- Smooth the segments at the turning areas as shown in Fig. 6. $\forall node\in E_{Turn}$, adjust the timing as follows:
  16 $$\begin{aligned} {\left\{ \begin{array}{ll} \Delta x=r\tan \left( \frac{\alpha }{2} \right) \\ \Delta {\tilde{s}}=\left| \frac{\alpha }{2} \right| r\\ t_{Turn}\prime =t_{Turn}-\frac{2\left( \Delta x-\Delta {\tilde{s}} \right) }{v_{Turn}}\\ \end{array}\right. } \end{aligned}$$
  Where $\alpha$ is the turning angle, *r* is the turning radius, $\Delta x$ is the advance distance for the turn, $\Delta {\tilde{s}}$ is half of the arc length corresponding to the turning angle, and $t_{Turn}^{\prime }$ is the adjusted timing for passing the turning point.
- Output the guidance trajectory:${\varvec{T}}\left\{ i,j \right\} =\left\{ \left( x_n,y_n,v_n,t_n \right) \left| n=0,1,2,\ldots ,t \right. \right\}$.

The guidance trajectory ${\varvec{T}}\left\{ i,j \right\}$ provides information about the working time of UFMC:

17 $$\begin{aligned} t_{ij}=t_t-t_0, \forall i\in G, j\in P \end{aligned}$$

Moreover, the guidance trajectory ${\varvec{T}}$ reflects the situation of the UFMC on surface, and serves as the foundation for the conflict prediction model.

#### Conflict prediction

A conflict prediction model based on protected zones is established by using the coordinates of each position in the trajectory as the geometric centers. The properties of a protected zone are determined by trajectory’s parameters of corresponding position, enabling the prediction of conflicts. Among commonly used protected zones, the elliptical demonstrates good prediction accuracy and intuitiveness.

**Figure 7:**
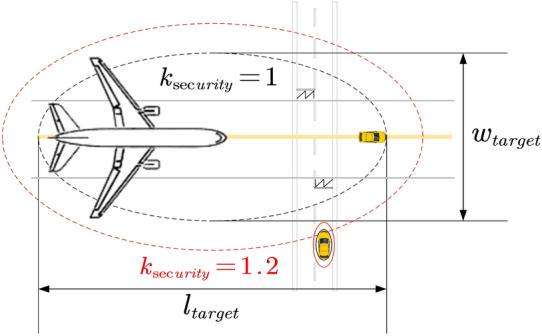
Schematic diagram of a protected zone.

The lengths of the semi-major axis “*a*” and the semi-minor axis “*b*” of the elliptical protected zone are calculated as following:

18 $$\begin{aligned} {\left\{ \begin{array}{ll} a=\left( \frac{l_{target}}{2}+\frac{v^2}{2a_d} \right) \cdot k_{\sec urity}\\ b=\left( \frac{w_{target}}{2} \right) \cdot k_{\sec urity}\\ \end{array}\right. } \end{aligned}$$

Here, $k_{\sec urity}$ represents the safety factor. $l_{target}$ and $w_{target}$ denote the length and width of the UFMC or GU, respectively. During the Guidance phase, the moving target refers to the GU. During the Dispatch or Recycle phase, the target refers to the UFMC:

19 $$\begin{aligned} l_{target}= & {} {\left\{ \begin{array}{ll} l_V+l_A+\Delta {\overline{L}}&{}\quad \text {if target is a Guidance Unit}\\ l_V&{} \quad \text {else}\\ \end{array}\right. }\end{aligned}$$

20 $$\begin{aligned} w_{target}= & {} {\left\{ \begin{array}{ll} w_A&{}\quad \text {if target is a Guidance Unit}\\ w_V&{}\quad \text {else}\\ \end{array}\right. } \end{aligned}$$

Here, $w_A$ represents the wingspan of the guided aircraft, and $w_V$ represents the width of the UFMC.

The purpose of setting the safety factor $k_{\sec urity}$ is to minimize the possibilities of both false alarms and missed alerts in conflict prediction. Figure 7 shows the scenarios of different safety factors corresponding to the protected area.

The black protected zone represents a safety factor of 1, which cannot envelop the entire moving target most of the time, potentially leading to missed alerts. The red protected zone corresponds to a safety factor of 1.2, which can envelop the entire moving target and minimize the probability of false alarms when potential conflicts are predicted. The Reference^24^ has already discussed incidents of wingtip collisions on the apron, it is sufficient for their model to recognize and deal with such incidents. Therefore, our model eliminates the wingtip clearance $\varepsilon$ proposed by Giuseppe S [7], and uses the length of the GU instead of the length of the aircraft in their tractor problem to ensure that there is sufficient space for our GU within the protected zone.

**Figure 8:**
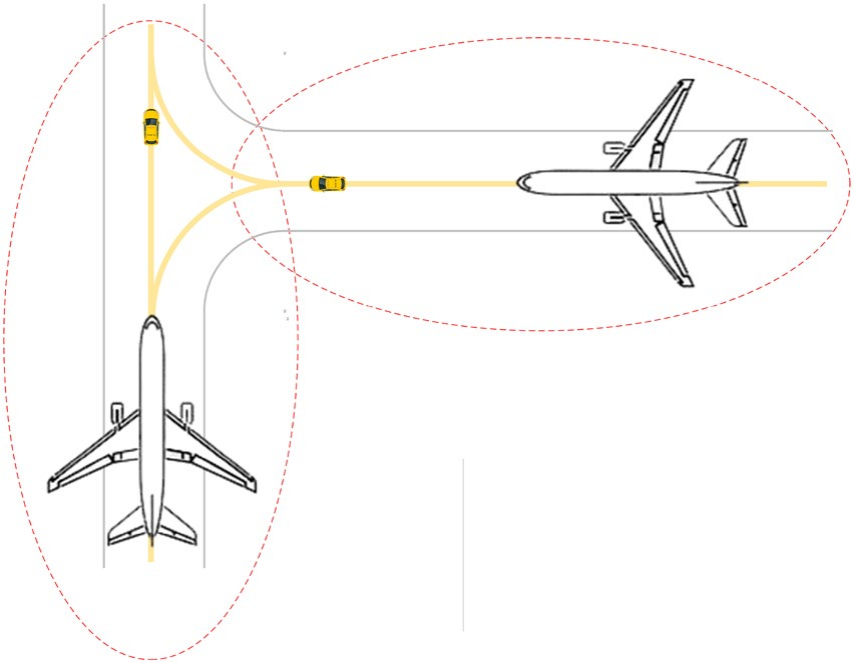
Schematic diagram of a typical situation of conflict.

Figure 8 visually shows a typical scenario of conflict between GUs. The elliptical protected zone could accurately represent the spatial occupation of GU, and maintain safe spacing both longitudinally and laterally. To reproduce the protected zone, and determine whether the ellipses overlap or not, We have formulated the elliptical equation as following:

21 $$\begin{aligned} \left( \frac{\left( x-x_n \right) \sin \theta -\left( y-y_n \right) \cos \theta }{a} \right) ^2+\left( \frac{\left( x-x_n \right) \cos \theta +\left( y-y_n \right) \sin \theta }{b} \right) ^2=1 \end{aligned}$$

Subsequently, the Algorithm 2 is used to output the number of conflicts, denoted as $N_{Conflict}$, thereby completing the conflict prediction.

**Algorithm 2:**
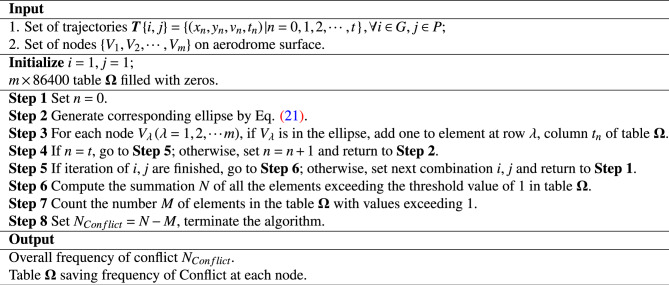
Conflict Prediction Algorithm.

### Model for UFMC assignment

**Figure 9:**
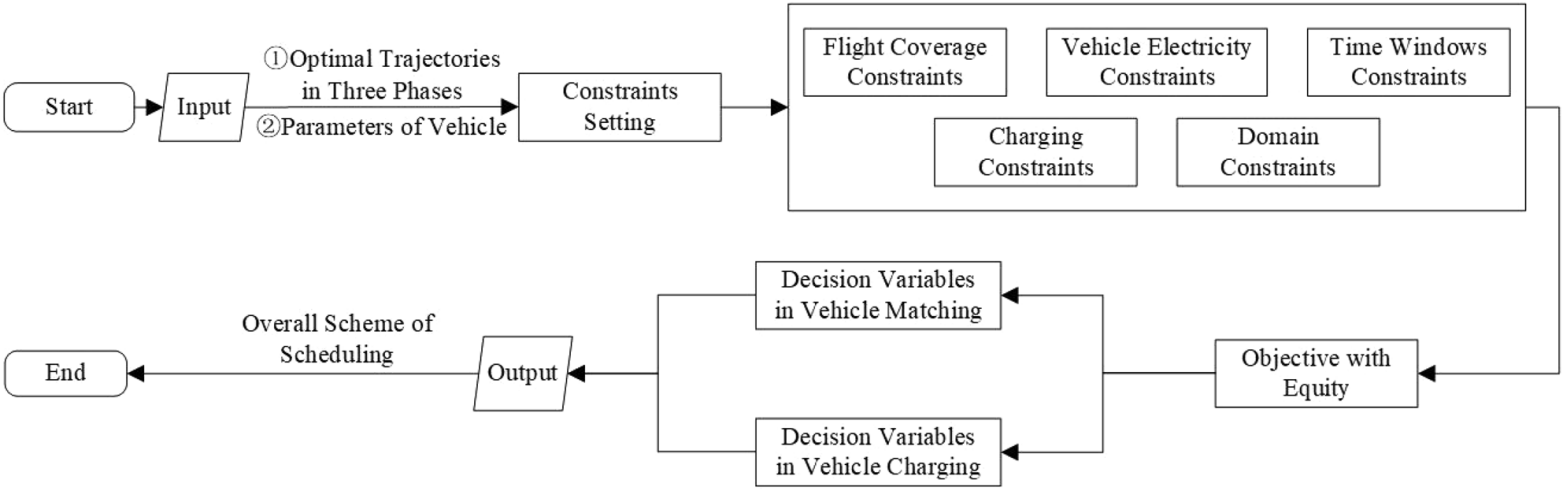
Framework of the model of UFMC Assignment.

Compared to traditional manually-operated vehicles that rely on human experience for scheduling, operation, and maintenance, UFMCs overcome the limitations of manual driving through fully automated control. Building upon precise trajectory planning, further considerations for vehicle maintenance and optimal allocation of vehicle resources are necessary. In this regard, an UFMC assignment model shown in Fig. 9 is designed in the UFMC scheduling system, wherein the optimal solution set of trajectories is transformed into specific guidance tasks, to achieved the function of assignment through ILP.

Considering that the source assignment problem can attribute to 0-1 programming. According to the general method in management of civil aviation^13^, we designed a model for vehicle assignment as following:

$$\begin{aligned} \min G=\frac{k+1}{k}-\frac{2}{k}\sum _{i=1}^s{\frac{\sum _{l=1}^k{\left( k-l+1 \right) x_{il}}}{\sum _{j=1}^3{\left( t_{ij}+\eta \right) }}} \end{aligned}$$

s.t.

$$\begin{aligned}{} & {} \sum _{l=1}^k{x_{il}}=1, \quad \forall i\in G\\{} & {} x_{ol}+x_{pl}\leqslant 1, \quad \forall l\in F\\{} & {} {\left\{ \begin{array}{ll} a_o\leqslant a_p+\eta +\sum _{j=1}^3{t_{pj}}\\ a_o+\eta +\sum _{j=1}^3{t_{oj}}\geqslant a_p\\ \end{array}\right. }\\{} & {} {\left\{ \begin{array}{ll} q\sum _{i=1}^s{d_{il}}-\sum _{i=1}^{i^*}{\left( \left( \sum _{j=1}^3{t_{ij}} \right) +\eta \right) x_{il}}\geqslant 0\\ q\sum _{i=1}^s{d_{il}}-\sum _{i=1}^{i^*}{\left( \left( \sum _{j=1}^3{t_{ij}} \right) +\eta \right) x_{il}}\leqslant q+q_{reserve}\\ \end{array}\right. }, \forall i^*\in G,l\in F\\{} & {} x_{ol}+x_{rl}\leqslant 2-d_{ol}, \forall l\in F\\{} & {} {\left\{ \begin{array}{ll} a_o\leqslant a_r+\eta +C+\sum _{j=1}^3{t_{rj}}\\ a_o+\eta +C+\sum _{j=1}^3{t_{oj}}\geqslant a_r\\ \end{array}\right. }, \forall l\in \complement _F\left\{ f_k \right\} \\{} & {} x_{il}={\left\{ \begin{array}{ll} 1&{}\quad \text {if vehicle } l\text { guides flight } i\\ 0&{}\quad \text {else}\\ \end{array}\right. }\\{} & {} d_{il}={\left\{ \begin{array}{ll} 1&{}\quad \text {if vehicle } l\text { is charged after flight } i\\ 0&{}\quad \text {else}\\ \end{array}\right. }\\{} & {} x_{il}-d_{il}\geqslant 0, \forall i\in G,l\in F \end{aligned}$$

#### Model assumptions

- The UFMCs at the end of the Recycle phase must return to the parking lot where they started, in order to perform the next guidance task.
- A buffer time of $\eta$ is allocated between two guidance tasks to ensure orderly traffic flow in and out of the parking lot, or transitioning to the corresponding parking lot for the next guidance task.
- A vehicle work continuously until its battery level reaches a low state, then return to the parking lot for a full recharge. The charging time *C* and the safe endurance *q* are fixed values specific to each type of vehicle. Factors such as battery decay and seasonal variations are ignored, but the buffer time $\eta$ is included in the continuous working time.

Let $F=\left\{ f_1,f_2,\ldots ,f_k \right\}$ represent the set of *k* UFMCs in the airport.

#### Objective function

This model aims to achieve the most equitable vehicle assignment, so we adopt the Gini coefficient as the evaluation metric. In the field of economics, a Gini coefficient between 0.3 and 0.4 is considered fair^25^. However, considering that the working time of UFMCs is a “negative asset”, the Gini coefficient is referred to as the “assignment inequality index” in this context. Therefore, the objective function is set to minimize the assignment inequality index of the working time for UFMCs:

22 $$\begin{aligned} \min G= & {} \sum _{l=1}^k{P_lY_l}+2\sum _{l=1}^k{P_l\left( 1-V_l \right) }-1\nonumber \\= & {} \frac{k+1}{k}-\frac{2}{k}\sum _{i=1}^s{\frac{\sum _{l=1}^k{\left( k-l+1 \right) x_{il}}}{\sum _{j=1}^3{\left( t_{ij}+\eta \right) }}} \end{aligned}$$

Where $P_l=\frac{1}{k}$, and $Y_l$ represents the proportion of working time for the *l*-th vehicle to the total working time, and $V_l$ is the cumulative distribution of $Y_l$. $\sum _{j=1}^3{t_{ij}}$ denotes the accumulated time for the three phases of a single guidance task. $x_{il}$ is a binary decision variable indicating whether vehicle *l* guides flight *i*.

#### Constraints

- Constraints of flight coverage

Each flight should be assigned to one UFMC ONLY:

23 $$\begin{aligned} \sum _{l=1}^k{x_{il}}=1,\quad \forall i\in G \end{aligned}$$

- Constraints of consecutive guidance tasks

The time windows of guidance tasks assigned to the same vehicle should not overlap. Let *o* and *p* represent a pair of guidance tasks corresponding to flights where their time windows overlap each others, then the constraints can be formulated as follows:

24 $$\begin{aligned} x_{ol}+x_{pl}\leqslant 1, \quad \forall l\in F \end{aligned}$$

The condition that the time windows of guidance tasks *o* and *p* overlap can be expressed as follows:

25 $$\begin{aligned} {\left\{ \begin{array}{ll} a_o\leqslant a_p+\eta +\sum _{j=1}^3{t_{pj}}\\ a_o+\eta +\sum _{j=1}^3{t_{oj}}\geqslant a_p\\ \end{array}\right. } \end{aligned}$$

Here, $a_o$ and $a_p$ correspond to the actual delivery times (ADT) of guidance tasks for flights *o* and *p*, respectively.

**Figure 10:**
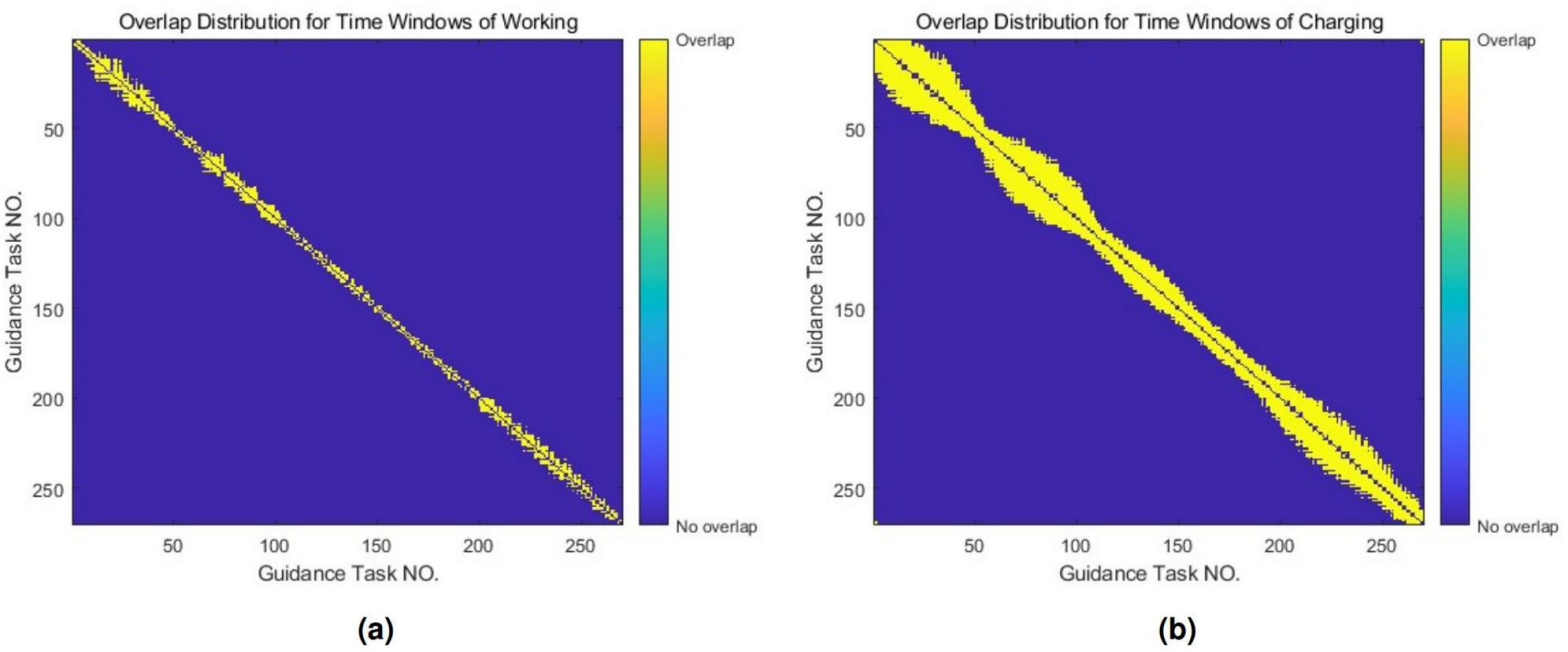
Overlap contribution. **(a)** For time windows of working. **(b)** For time windows of charging.

Usually, guidance tasks are sorted by ADT. Fig. 10a shows the overlap situation of time windows between adjacent index of guidance tasks.

- Constraints of vehicle electricity

Constraints of vehicle electricity ensure that the quantity of electricity of the UFMCs meets both upper and lower limit requirements during operation and forms a closed loop for charging:

26 $$\begin{aligned}{} & {} {\left\{ \begin{array}{ll} q\sum _{i=1}^s{d_{il}}-\sum _{i=1}^{i^*}{\left( \left( \sum _{j=1}^3{t_{ij}} \right) +\eta \right) x_{il}}\geqslant 0\\ q\sum _{i=1}^s{d_{il}}-\sum _{i=1}^{i^*}{\left( \left( \sum _{j=1}^3{t_{ij}} \right) +\eta \right) x_{il}}\leqslant q+q_{reserve}\\ \end{array}\right. }\nonumber \\{} & {} \quad , \forall i^*\in G,l\in F \end{aligned}$$

Here, $d_{il}$ is the binary representing whether vehicle *l* requires charging after completing task *i*. $q_{reserve}$ is a backup of the quantity of electricity that ensures the UFMC can safely return to the parking lot after operating for a continuous duration of *q*.

- Constraints of charging

For single vehicle, the guidance task should not have conflict with the charging process:

27 $$\begin{aligned} x_{ol}+x_{rl}\leqslant 2-d_{ol},\quad \forall l\in F \end{aligned}$$

The conflict condition between the time windows of guidance tasks *o* and *r* can be expressed as follows:

28 $$\begin{aligned} {\left\{ \begin{array}{ll} a_o\leqslant a_r+\eta +C+\sum _{j=1}^3{t_{rj}}\\ a_o+\eta +C+\sum _{j=1}^3{t_{oj}}\geqslant a_r\\ \end{array}\right. } \end{aligned}$$

Figure 10b shows the overlap situation between the time windows of a guidance task with charging demand, and its follow-up tasks.

- Constraints of vehicle sorting

**Figure 11:**
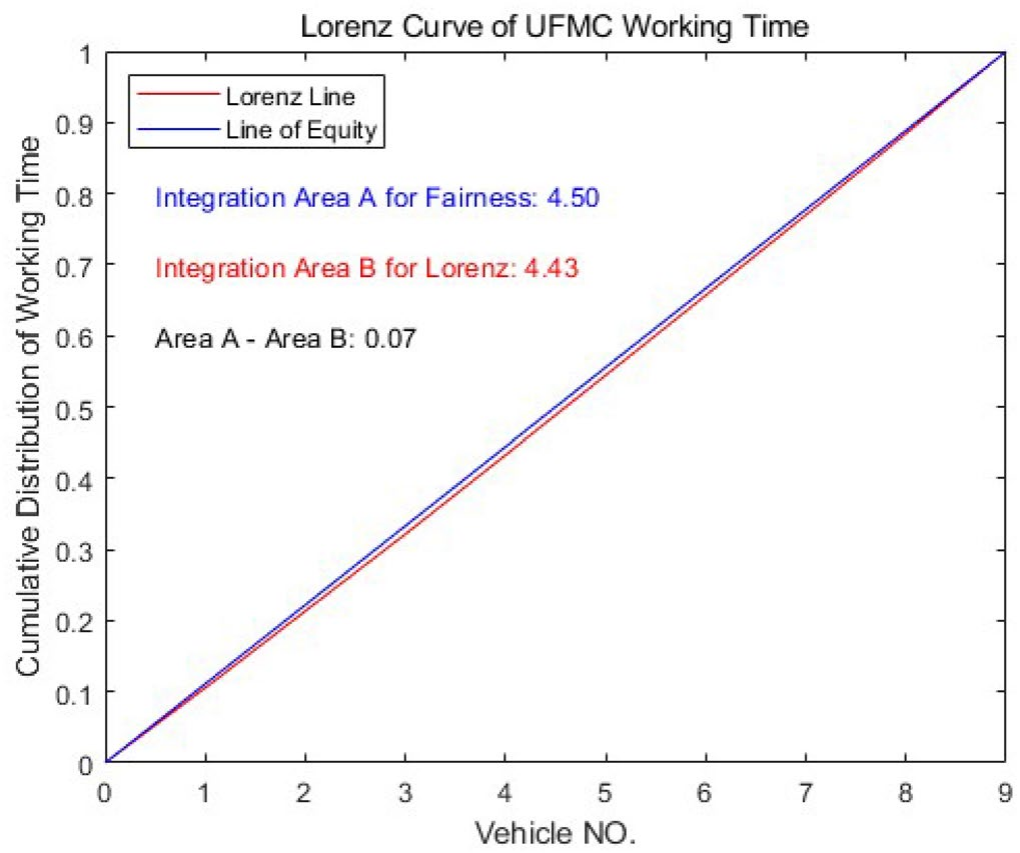
Calculation of assignment inequity index with equity curve and Lorenz curve.

As Fig. 11 is shown, the calculation of assignment inequity index is related to the equity curve and the Lorenz curve. Both of them are formulated on the basis of cumulative distribution of UFMC working time. Therefore, each element of the set of vehicles $F=\left\{ f_1,f_2,\ldots f_n \right\}$ should be sorted in ascending order based on its working time:

29 $$\begin{aligned}{} & {} \sum _{i=1}^s{\sum _{j=1}^3{\left( t_{ij}+\eta \right) }}x_{il}\leqslant \sum _{i=1}^s{\sum _{j=1}^3{\left( t_{ij}+\eta \right) }}x_{i\left( l+1 \right) }\nonumber \\{} & {} \quad , \forall l\in \complement _F\left\{ f_k \right\} \end{aligned}$$

- Constraints of domain

30 $$\begin{aligned}{} & {} x_{il}={\left\{ \begin{array}{ll} 1&{}\quad \text {if vehicle } l\text { guides flight }i\\ 0&{}\quad \text {else}\\ \end{array}\right. } \end{aligned}$$

31 $$\begin{aligned}{} & {} d_{il}={\left\{ \begin{array}{ll} 1&{}\quad \text {if vehicle }l\text {is charged after flight}\, i\\ 0&{}\quad \text {else}\\ \end{array}\right. } \end{aligned}$$

32 $$\begin{aligned}{} & {} x_{il}-d_{il}\geqslant 0, \quad \forall i\in G,l\in F \end{aligned}$$

## Solution algorithm

In this section, we design corresponding solution algorithm based on characteristics of models mentioned, and construct an overall solution framework for the scheduling problem for UFMCs.

**Figure 12:**
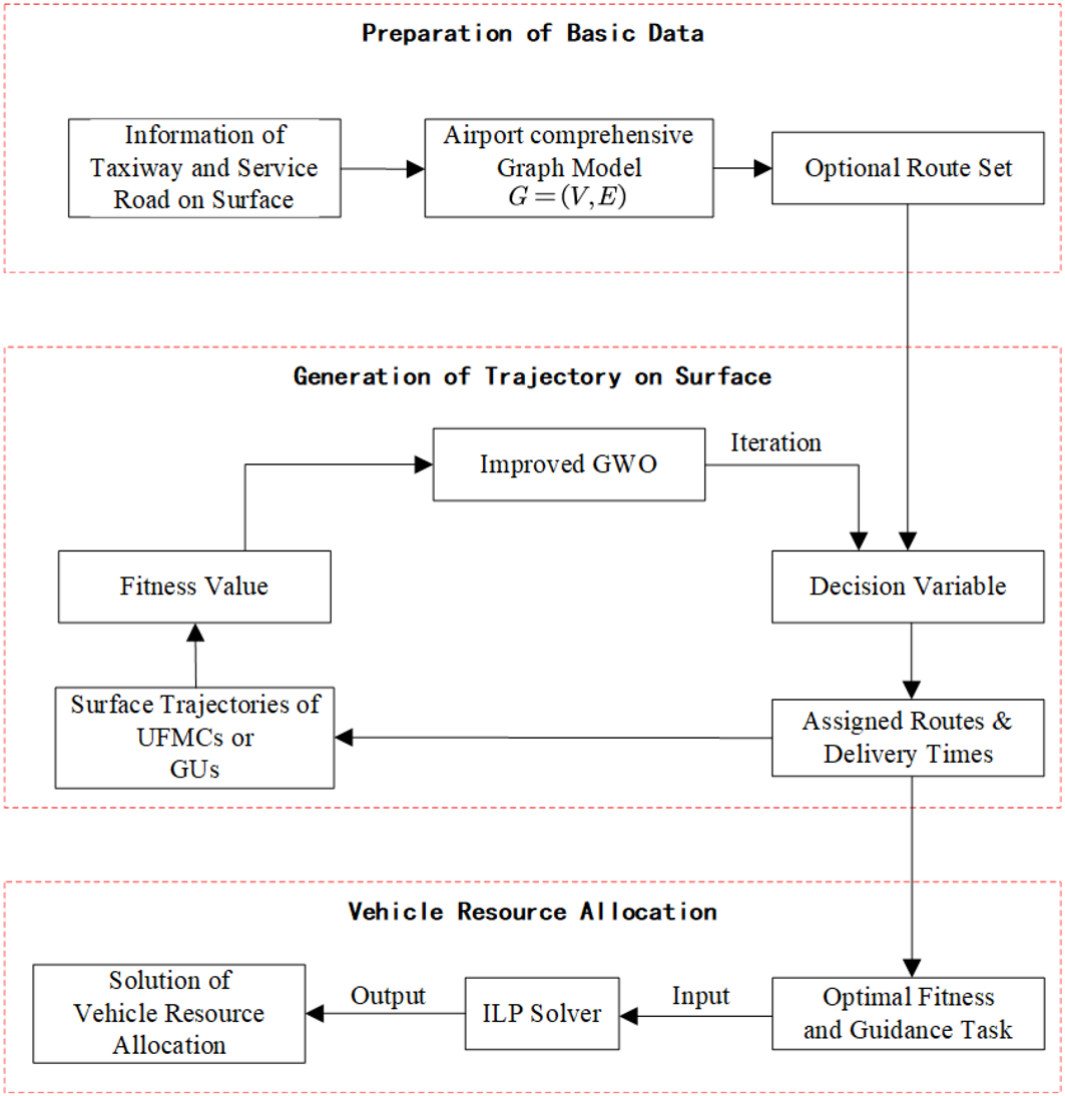
Framework of solution method.

As Fig. 12 is shown, the solution process for the scheduling model for UFMCs consists of three steps: preparation of basic data, generation of trajectory on surface, and vehicle resource allocation.

- In the step of basic data preparation, a comprehensive graph model $G=\left( V,E \right)$ is generated, where the edges *E* correspond to various road sections in the scene, including $E_{Apron}$ (the service roads, taxiways and taxi links within the apron), $E_{Taxiway}$, taxiways in the maneuvering area, as well as $E_{Turn}$, the various intersections and turns. The vertices *V* represent the endpoints of the edges, including key points like turning points, entrances/exits, and crossings. Then, generate a set of candidate routes for each pair of origins and destinations (ODs) on airport surface^26^.
- In the step of generation of trajectory on surface, the decision variables $\left\{ \left( R_{ij},\gamma _{ij} \right) \left| i\in G, j\in P \right. \right\}$ are transformed into the assigned routes and delivery times in three phases of each guidance task. A function of fitness is formed by integrating the collaborative planning model for surface guidance trajectories and the conflict prediction model based on protection zones. The optimal set of surface guidance trajectories are formed iteratively by the IGWO algorithm.
- In the step of vehicle resource allocation, the optimal set serves as the input for the UFMC assignment model. The model is solved directly using a solver to output the allocation scheme for vehicle resource.

The complexity analysis shows that the scale of feasible solution of the collaborative planning model for surface guidance trajectories is extremely large, with $2^s$ possible combinations of ONLY the route components. When the number of daily flights exceeds 50, there is likely to be a combinatorial explosion problem. The scale of the decision variable for the UFMC assignment problem is $\left( 2ns \right)$, which is a relatively manageable classic ILP problem and does not require a heuristic algorithm additionally.

Therefore, to address the collaborative planning model for surface guidance trajectories, an IGWO algorithm based on integer encoding is designed to improve the efficiency of generation for the optimal set of surface trajectories.

### Encoding strategy and initialization

When we apply this algorithm, it is necessary to define the meaning of each dimension in the position vector of a grey wolf individual. Based on the decision variables of the collaborative planning model, targeting guidance tasks for *s* flights, we construct an integer space with 6*s* dimensions as shown in Table 2.

**Table 2:** The vector of grey golf ${\varvec{X}}$ with integer encoding.

	$g_1$	$g_2$	$\ldots$	$g_s$	Explanation
${\varvec{X}}_{i1}$	1	1	$\ldots$	1	Route NO. at Phase 1
${\varvec{X}}_{i2}$	2	1	$\ldots$	1	Route NO. at Phase 2
${\varvec{X}}_{i3}$	1	2	$\ldots$	1	Route NO. at Phase 3
${\varvec{X}}_{i4}$	55	6	$\ldots$	45	Time adjustment at Phase 1
${\varvec{X}}_{i5}$	488	20	$\ldots$	271	Time adjustment at Phase 2
${\varvec{X}}_{i6}$	33	276	$\ldots$	54	Time adjustment at Phase 3

Considering that the UFMC scheduling system may avoid conflicts by adjusting routes, shifting time windows, or using a combination of both, conflict-free solution sets would be dispersed at multiple locations, we randomize the wolf pack initially, then integer by rounding to construct the integer space. This serves as the start for iteration and optimization.

### Fitness function

The calculation of the fitness function needs to consider the total working time on airport surface, which is based on the selected route, and needs to deduct the situation of potential conflict between the selected route and the variation in delivery time. Therefore, the fitness function *f* is defined as follows:

33 $$\begin{aligned} f= & {} \theta _1\left( 1-\frac{1}{\sum _{i=1}^s{\sum _{j=1}^3{t_{ij}}}} \right) \nonumber \\{} & {} +\theta _2\sum _{t^*=1}^{86400}{\textbf{card}\left( node\in \left\{ Ellipses_{t^*} \right\} \right) } \end{aligned}$$

In this equation, the first term, which relates to the target parking number and selected route, can be obtained by from the pre-computed values of $t_{ij}$ based on the given timetable of flights and information of grey wolves. The second term requires traversing each unit of time for movement on the surface, and $\forall node$,we need to count the number of times a node falls in different ellipses.

### Enhancing global search

In the local search, the slight decimal-level changes in the positions of the wolf pack in the traditional GWO algorithm are not suitable for integer encoding, so we adopt the IGWO algorithm proposed by Yang Z^27^ to enhance the prominence and efficiency of global search during the iteration process. The formula for updating the convergence factor is given as follows:

34 $$\begin{aligned} a^{\prime }=2e^{-\frac{t}{T}} \end{aligned}$$

Where the improved convergence factor $a^{\prime }$ starts from the default value of 2 and exponentially decays to $\frac{2}{e}$ with the iteration process. When approximately 69.3 percents of the iteration process is completed, the improved algorithm enters the local search, and the global-to-local search ratio shifts from 1:1 to 7:3.

### Improvement for local search

For the local search, we adopt a probabilistic method for updating position^28^:

35 $$\begin{aligned} {\varvec{X}}\left( t+1 \right) = {\left\{ \begin{array}{ll} {\varvec{X}}_1\,\,r_c\in \left( 0,\frac{1}{3} \right] \\ {\varvec{X}}_2\,\,r_c\in \left( \frac{1}{3},\frac{2}{3} \right] \\ {\varvec{X}}_3\,\,r_c\in \left( \frac{2}{3},1 \right] \\ \end{array}\right. } \end{aligned}$$

Here, $r_c$ is a random number that satisfies $\textrm{U}\left( 0,1 \right)$. The formula for updating position based on integer space not only reflects the dynamic process of the wolf pack following the leader, but also ensures the meaningfulness of the operation of updating position for ${\varvec{X}}\left( t+1 \right)$ in the local search.

### IGWO algorithm

In summary, the flow of the IGWO algorithm is shown as following:

**Algorithm 3:**
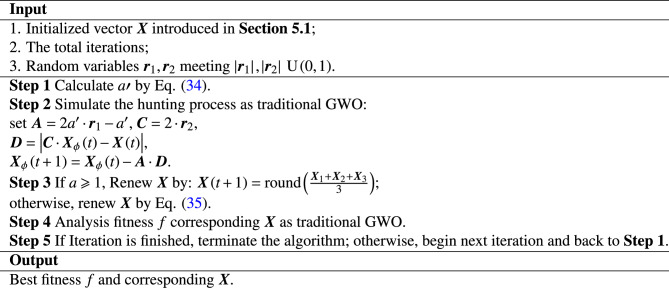
IGWO Prediction.

## Preparation of data

Ezhou Huahu Airport is a specific cargo airport in China, it is also an important experiment field for unmanned ground support vehicles, but there is a lack of overall scheduling for large-scale fleet.Taking Ezhou Huahu Airport for simulation experiment, we investigate the optimization of scheduling for UFMCs during a typical working day in 2030, with 270 flights per day, and assume that runway-to-stand assignments are based on the principle of proximity.

**Figure 13:**
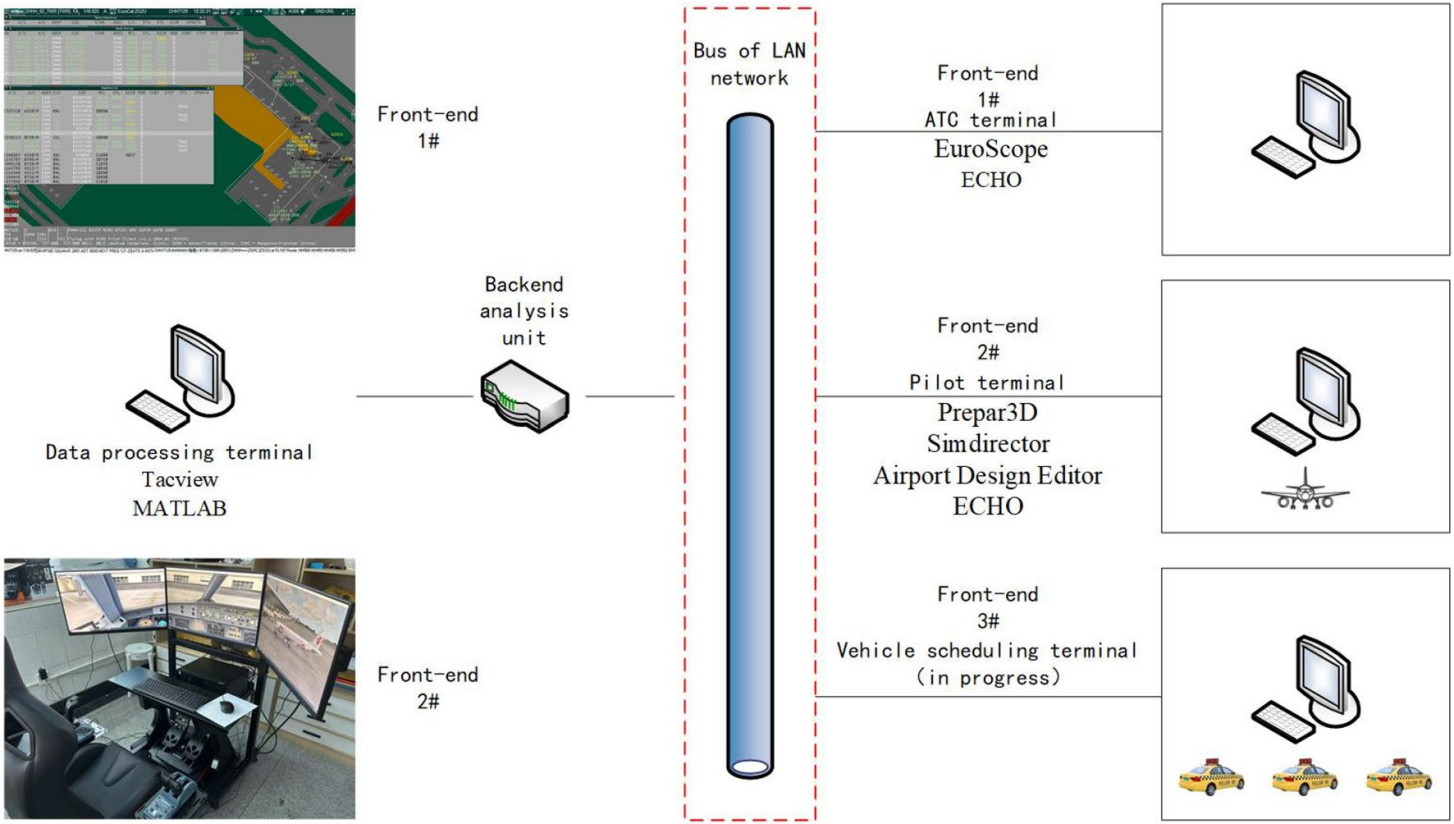
Schematic diagram of the UFMC’s routes.

Before starting the experiment, we has built a simulation platform. It is divided into air traffic control (ATC) terminal, pilot terminal, and data processing terminal. The interaction between terminals, and the details about hardware and software are shown in Fig. 13.

### Basic routes

**Figure 14:**
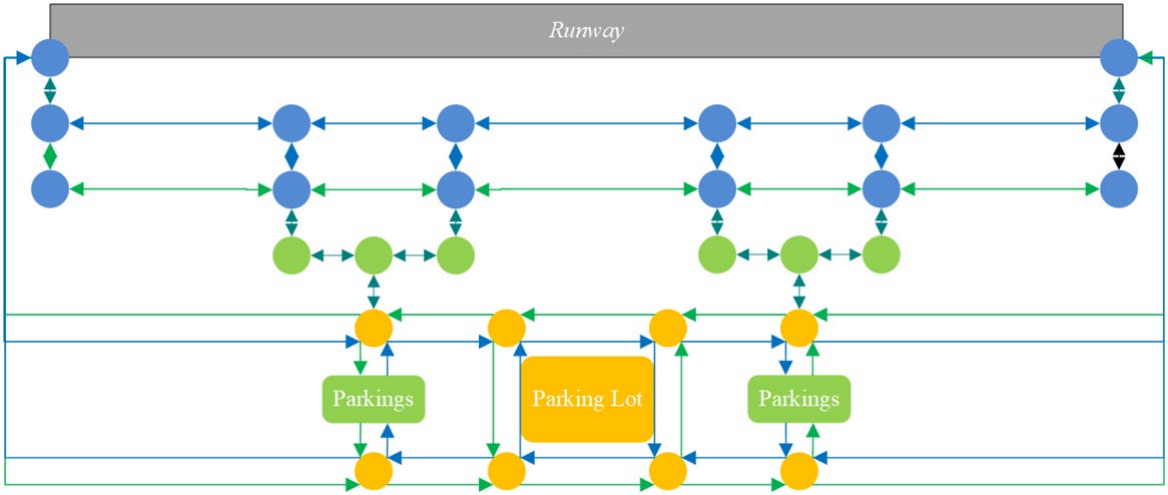
Schematic diagram of the UFMC’s routes.

Figure 14 shows the schematic diagram of the UFMC’s routes. During phases 1 and 3 (operations on service roads), the strategy of separating inner and outer loops is employed. In phase 2 (on-taxiway) operations, the “fixed, one-way, directional, cyclic” principle^29^ is followed, where alternative routes are assigned to vehicles, to prevent and alleviate congestion, and to minimize the occurrences of conflict initially. The number of alternative routes, denoted as *k*, is set to 2. The blue and green arrows in the figure represent Route 1 and Route 2, respectively.

**Figure 15:**
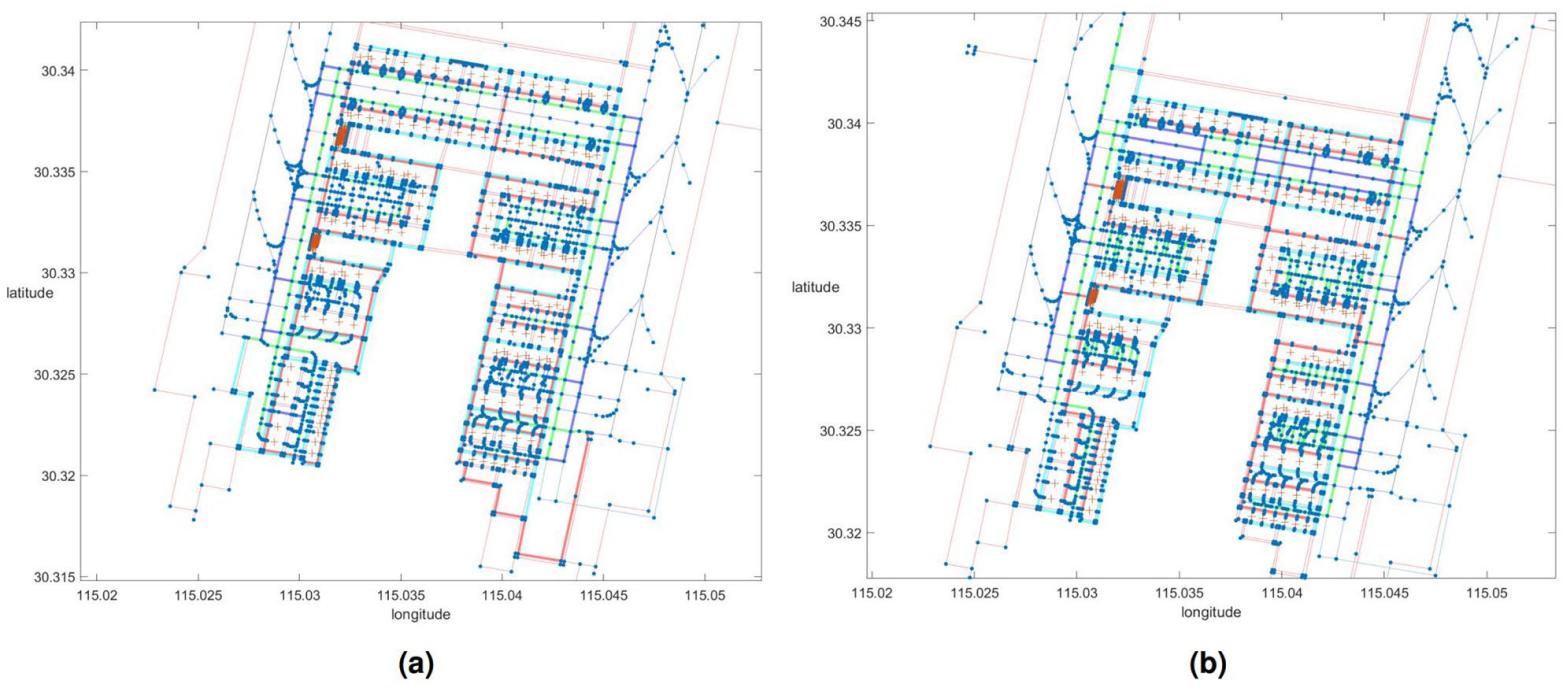
Schematic diagram of optional route set for Ezhou Airport. **(a)** Route set for Departure. **(b)** Route set for Arrival.

Based on the strategy for route allocation, Ezhou Airport’s UFMCs have a set of selectable routes shown in Fig. 15. Figure 15a and b show the departure and arrival scenarios, respectively. In these figures, Route 1 is highlighted in blue (shared with Route 2 in green) during the Guidance phase, while Route 2 is labeled in green. The Dispatch and Recycle phases are marked by the red and cyan lines for Route 1 and 2, respectively.

### Parameter settings

According to the actual operation of Ezhou Airport and the requirements of Chinese regulations [20], the parameters of the UFMC are shown in Table 3. The UFMC is allowed to arrive in advance and wait for a maximum of 60s. The time windows for air traffic controllers to adjust the ETD and ETA of flights are 600 s and 300 s, respectively. Therefore, in the case of arrival, the upper limits of the change in delivery times for UFMCs ($\gamma _{i1}$ to $\gamma _{i3}$) are 60s, 300s, and 60s, respectively; for departure, those are 60s, 60s, and 600s, respectively.

**Table 3:** Parameter settings for the fleet of UFMCs.

Parameter	Explanation	Value	Parameter	Explanation	Value
*q*(h)	Safety endurance	12	$a_a[\hbox {m/s}^{2}]$	Acceleration	1
$q_{reserve}$(min)	Reserve endurance	15	$a_d[\hbox {m/s}^{2}]$	Deceleration	1
$v_{Taxiway}$(m/s)	Velocity on main taxiways	10	$l_{car}$(m),$w_{car}$(m)	Length and width	4.8, 1.8
$v_{Apron}$(m/s)	Velocity in apron	8	*C*(min)	Required Time for Charging	45
$v_{turn}$(m/s)	Velocity at turns	5	$\Delta {\overline{L}}$(m)	Average Following Space	60

### Profiles generation

**Figure 16:**
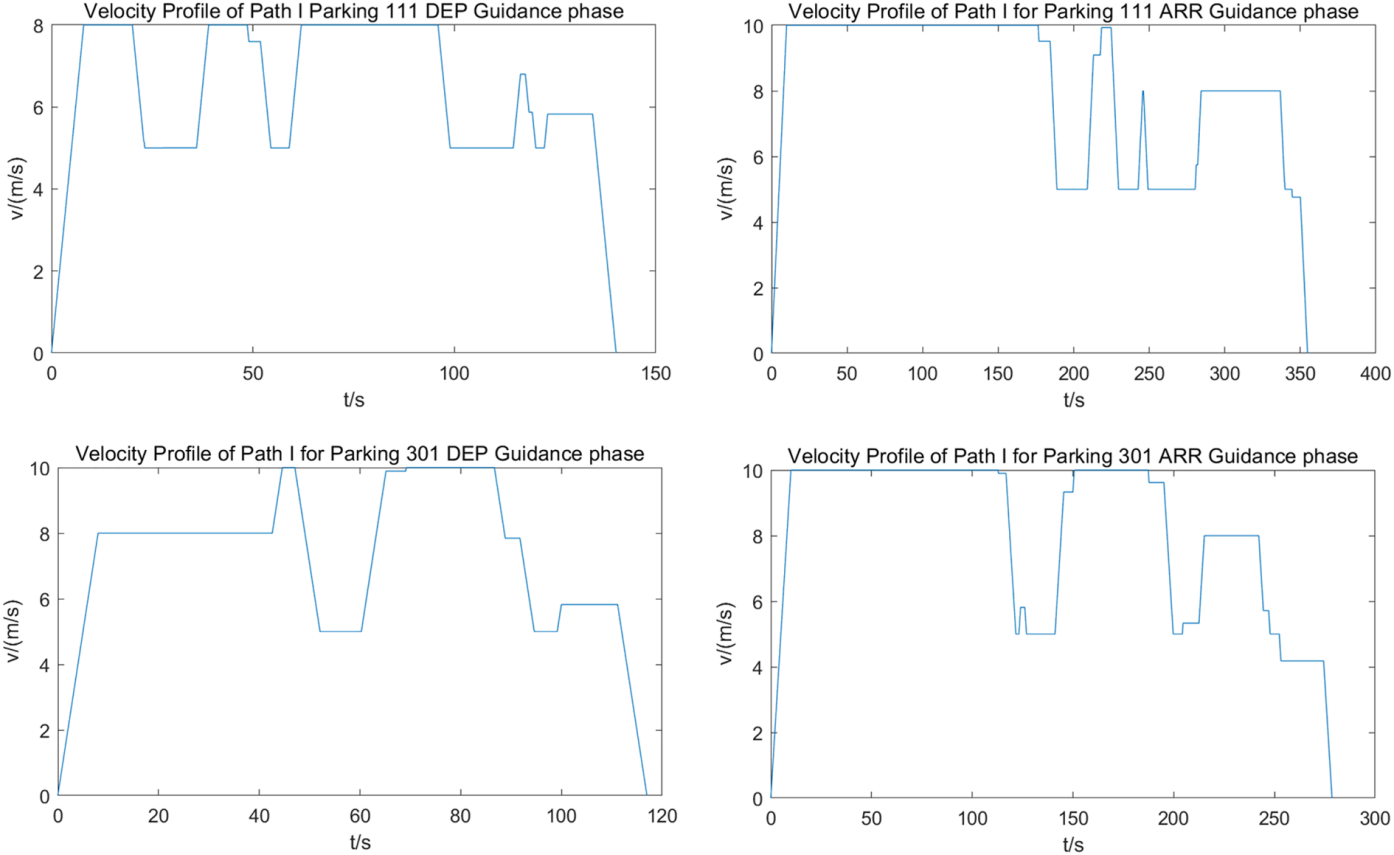
The velocity profiles along typical routes.

To illustrate this process, we selected Route 1, along with guarded flights at Stand 111 and 301 as examples, and generated velocity profiles by Algorithm 1. Figure 16 shows the typical velocity profiles.

In Fig. 16, the velocity of the GU remains stable at 5 m/s, 8 m/s, and 10 m/s, corresponding to turns, aprons, and straight sections of taxiways in maneuvering areas, respectively. As shown in the second half of each profile, the UFMC adjusts its cruising speed, throttle, and brake, based on a comprehensive modification of the length of each segment and the initial settings, through the application of the Algorithm 1. The velocity is changed when the GU passes through different areas and segments, suggests that the efficiency of the UFMCs scheduling is related to the number of turns, taxiing distance, and timing of crossing the apron area, which confirms the importance of route selection in the trajectory planning module.

### Conflicts analysis

**Figure 17:**
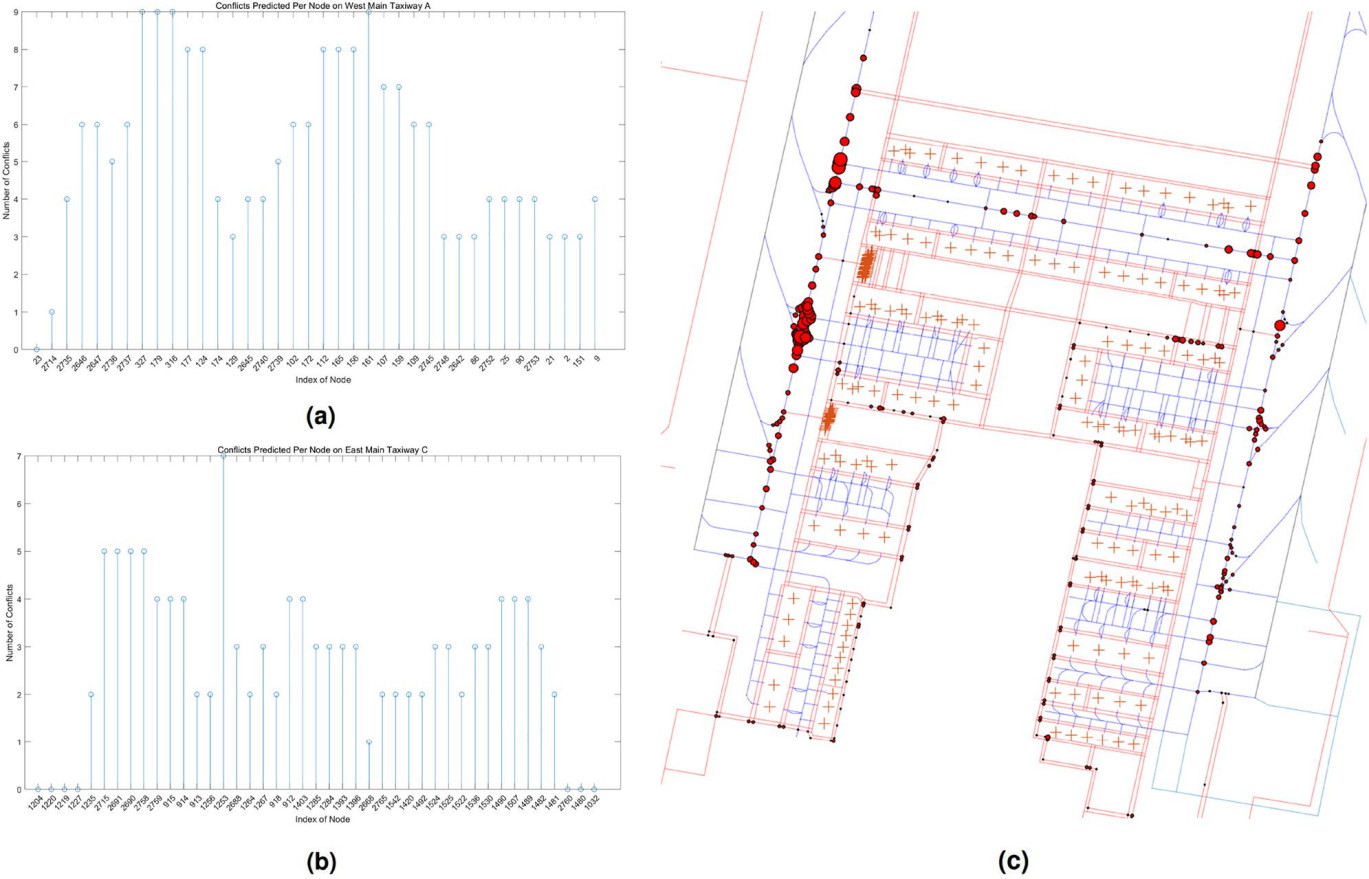
Distribution of conflicts before optimization.The southern end of the taxiway corresponds to the right-hand side of the horizontal axis. **(a)** Conflicts on the west main taxiway. **(b)** Conflicts on the east main taxiway. **(c)** Conflicts on airport surface.

An analysis of conflict characteristics before the optimization of scheduling was conducted. Without considering the selection of surface guidance trajectories and adjustments to delivery times, Algorithm 2 predicted 824 instances of conflicts among the trajectories. Figure 17a, b, and c show the characteristics distribution of conflicts spatially, for the west main taxiway, the entire surface, and the east main taxiway, respectively. The characteristic of “high in the north, low in the south” of the distribution of conflicts indicates that, when operating towards the north, the conflicts during arrival are higher than those during departure. On the other hand, due to the coexistence of passenger and cargo parking stands on the west side of Ezhou Airport, the distribution also exhibits the characteristic of “high in the west, low in the east”.

## Results and discussion

### Regards to vehicle trajectory planning

**Figure 18:**
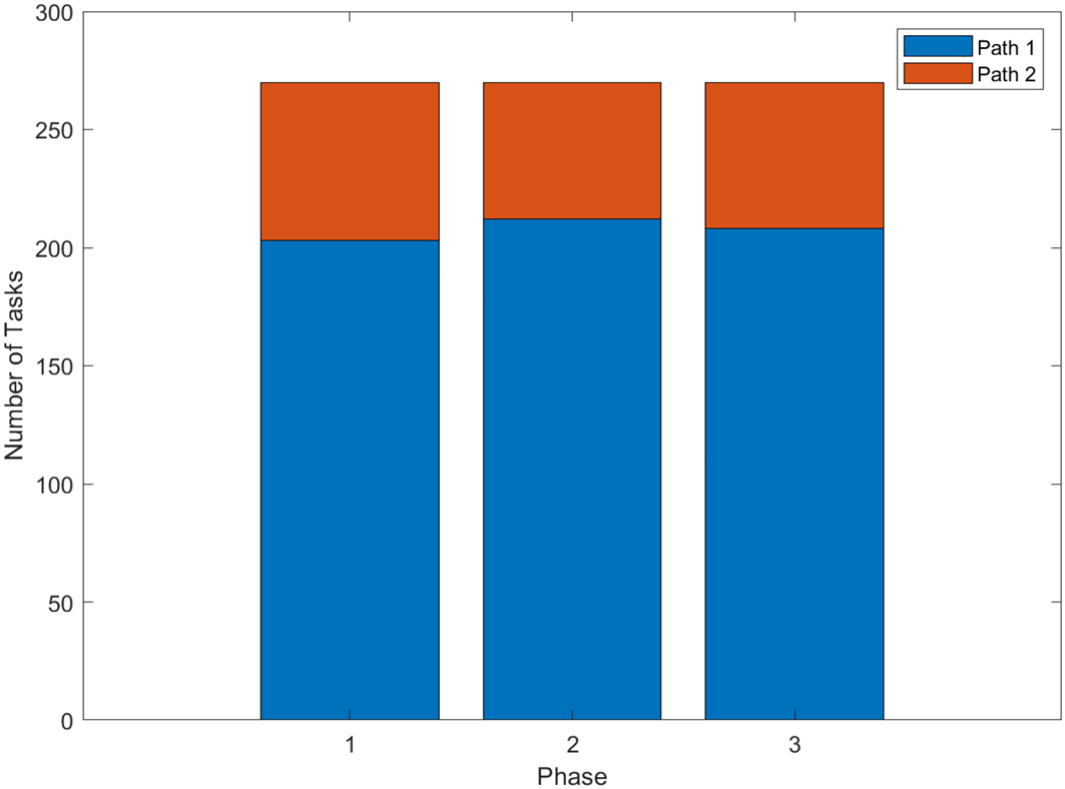
Histogram for frequency distribution of route selection.

After the computation of the vehicle trajectory planning module, the UFMC scheduling system generated a set of conflict-free guidance trajectories, with a total working time of 170,463s. The surface guidance trajectory is mainly planned collaboratively in three aspects: route selection, UFMC’s working time, and delivery sequence, achieving the effect of optimization. Among them, the distribution of route selection is shown in Fig. 18. The histogram indicates that Route 1 with shorter travel time remains the top choice for UFMCs. In phases 1 to 3, 24.81 %, 21.48 %, and 22.96 % of guidance tasks respectively choose Route 2 to avoid potential conflicts at the cost of detour.

**Figure 19:**
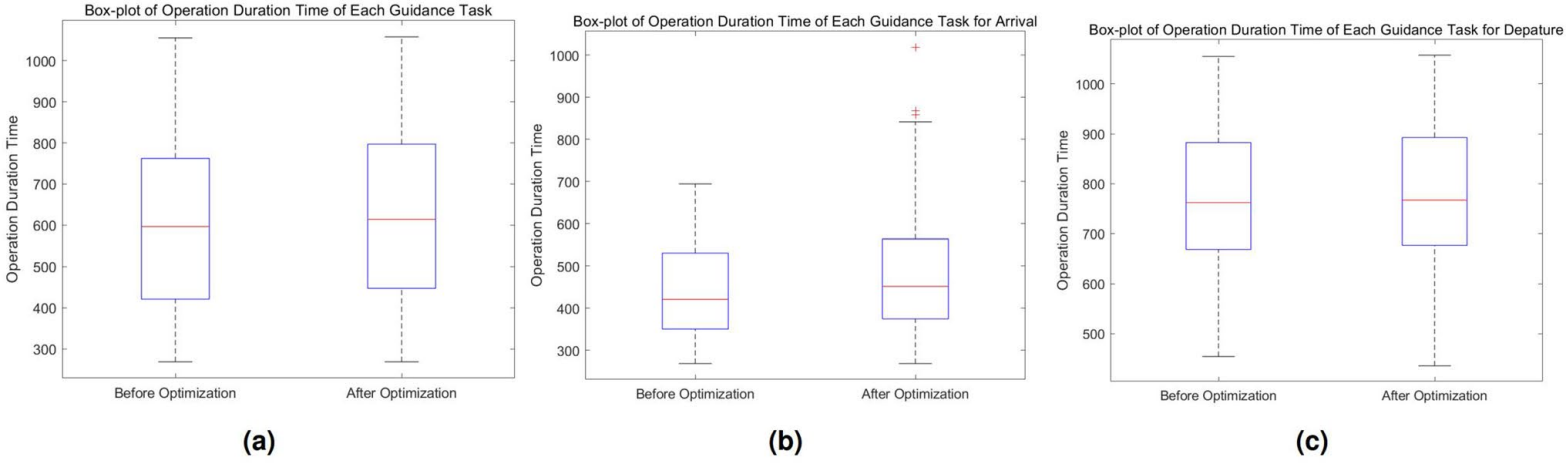
Box-plot of UFMC working time. **(a)** Overall distribution of working time. **(b)** Distribution of working time for arrival. **(c)** Distribution of working time for departure.

Adjustments in UFMC working time are shown in Fig. 19. Figure 19a shows that by extending the working time of UFMCs by 2.85%, the vehicle trajectory planning module achieves the resolution for all the conflicts. Figure 19b and c show that sacrificing operational efficiency is targeted. For arrival guidance tasks with higher conflict frequency, 7.15% of efficiency is sacrificed, while for departure, the change is minimal, with only a 0.66% extension of UFMC working time.

Regarding the result of delivery sequence shown in Table 4, a significant number of guide tasks have been adjusted by fine-tuning the delivery time to avoid conflicts. This has altered the regular delivery sequence, ensuring safety without the need for detours. Out of a total of 270 guide tasks, 64.44% have undergone adjustments in the delivery sequence.

**Table 4:** Result of delivery sequence.

Regular delivery sequence	Actual delivery sequence	Actual delivery time
1	**2**	0:07:57
2	**1**	0:07:36
3	3	0:15:14
$\dots$	$\cdots$	$\cdots$
39	**40**	2:06:41
40	**39**	2:05:48
41	**42**	2:24:17
42	**41**	2:14:52
43	**45**	2:35:36
44	**43**	2:34:02
45	**44**	2:35:10
46	**47**	2:44:54
$\cdots$	$\cdots$	$\cdots$
270	270	23:25:06

Significant values are in [bold].

### Regards to IGWO Algorithm

**Figure 20:**
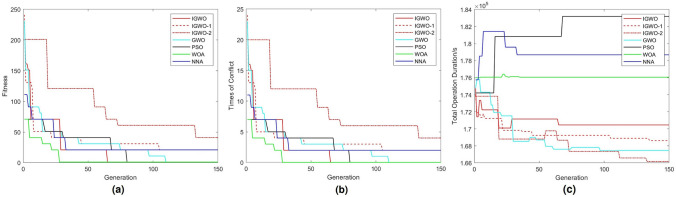
Convergence analysis of five algorithms. IGWO-1 ONLY incorporates the improvement defined by Eq. (34), while IGWO-2 employs Eq. (34) and Eq. (35) uniformly in both the global search and local search. **(a)** Fitness values. **(b)** Conflict counts. **(c)** Total working time of UFMCs.

To evaluate the convergence speed of the IGWO algorithm, seven different algorithms, including PSO, GWO, WOA^30^, NNA^11,31^, two variations of IGWO algorithm (IGWO-1 and IGWO-2), and the algorithm in this paper (IGWO), were employed in the case study. A population size of 20 and 150 iterations were set, the number of iterations is enough to to make the function variation converge within 1%. The results are showed in Fig. 20.

IGWO-1, IGWO-2 and NNA failed to generate any feasible conflict-free solution set for the scale of 270 flights. On the other hand, the remaining algorithms successfully produced feasible solution sets. Among the feasible, GWO exhibited the highest quality but had relatively slower convergence speed. Although PSO and WOA generated the feasible with ideal iterations, their total working times of UFMCs significantly deviated from that of the other algorithms, indicating inefficient operational performance. In contrast, IGWO showed the fastest convergence speed.

Furthermore, in Table 5, quantitative analyses were conducted on four major indicators for handling the scales of 12, 26, 83, 160, and 270 flights per day. In general, IGWO has got the most advantageous indicators (as shown in bold black in the table). WOA is competitive in the convergence speed, but performs poorly in the working time. In contrast, NNA seems to be adapted to small-scale problem.

**Table 5:** Quantitative assessment for algorithms. In the same experiment group, the black bold comments mean advantageous indicators.

Sorties of takeoff and landing	Algorithm	Rate of conflict resolution[%]	Required generation finding a feasible solution set[s]	Total working time of UFMCs[s]	Actual number of iterations
270	PSO	**100**	80	183,192	80
	GWO	**100**	110	**167,471**	110
	**IGWO**	**100**	**65**	**170,463**	**65**
	IGWO-1	91.67	$>150$	168,625	$>150$
	IGWO-2	80	$>150$	166,155	$>150$
	WOA	**100**	**28**	176,057	**35**
	NNA	81.82	$>150$	178,684	$>150$
12	PSO	100	Feasible Initially	6,843.1	**2**
	**IGWO**	100	Feasible Initially	**6,744.9**	18
	IGWO-1	100	Feasible Initially	6,801.2	**10**
	IGWO-2	100	Feasible Initially	6,788.9	32
	WOA	100	Feasible Initially	6,851.2	17
	NNA	100	Feasible Initially	**6,734.3**	149
26	PSO	100	**Feasible Initially**	15,157	**2**
	**IGWO**	100	3	**13,366**	149
	IGWO-1	100	3	13,632	89
	IGWO-2	100	38	13,478	137
	WOA	100	4	**13,232**	**22**
	NNA	100	**2**	14,357	119
83	PSO	100	**5**	52,513	**5**
	**IGWO**	100	18	**43,867**	145
	IGWO-1	100	**5**	**44,507**	**107**
	IGWO-2	100	67	45,219	149
	WOA	100	**3**	48,575	110
	NNA	100	79	49,665	143
160	PSO	**100**	**28**	104,807	**28**
	**IGWO**	**100**	44	98,233	**44**
	IGWO-1	**100**	89	**96,215**	89
	IGWO-2	50	$>150$	**96,531**	$>150$
	WOA	**100**	**6**	97,369	70
	NNA	**100**	44	102,518	123

Significant values are in [bold].

The performance of GWO, IGWO-1 and IGWO-2 showed improvements compared to the PSO algorithm mentioned in Reference^23^. In the case of 270 flights, the proposed IGWO algorithm achieved an 18.75% improvement in convergence speed, which is consistent with the qualitative analysis. Simultaneously, it incurred a relatively minor loss in operational efficiency, with only 1.76% more total working time compared to GWO. Comparative analysis across different scales revealed that IGWO exhibited strong generalization performance compared to the other algorithms. Meanwhile, IGWO-1 demonstrated potential advantages in solving medium-scale (83 and 160 flight movements) scheduling problems, showing promise for generating more efficient guidance trajectories.

### Regards to vehicle assignment

**Figure 21:**
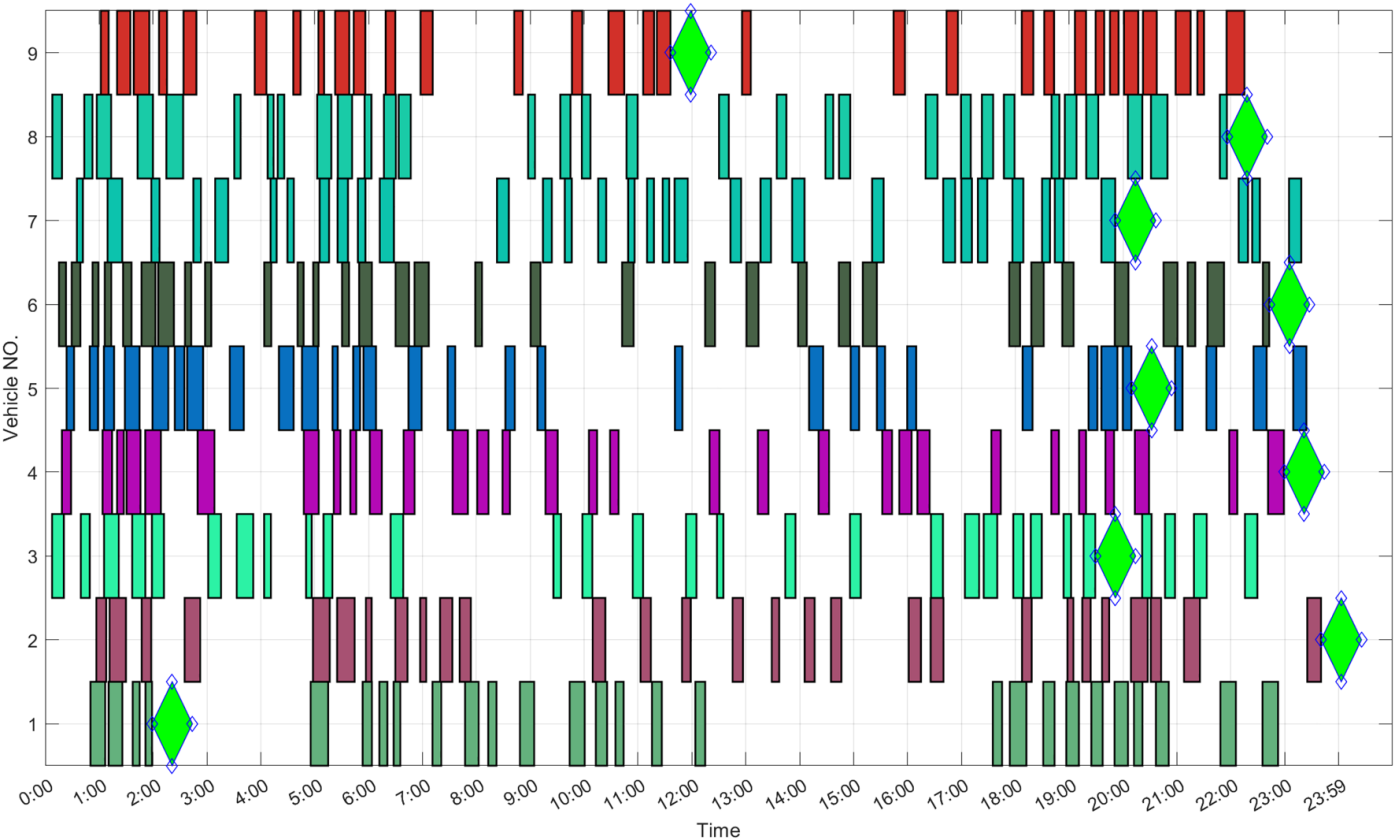
Gantt chart for guidance tasks and charging decisions. The green diamond represents the occurrence of a charging event.

According to the consultation notice^32^, the estimated number-*k* of UFMCs in this case study is 9. The results for optimization of UFMC assignment are shown in Fig. 21. The results indicate that each UFMC is assigned 28 to 34 flight tasks, with a standard deviation of 582s in working time. The assignment inequality index is only 0.015731, achieving fair vehicle assignment. Each UFMC only needs to be charged once per day, which is consistent with the total working time of 170,463s mentioned in Section 5.5. When 9 UFMCs are required, the average working time per vehicle is 18,940s, less than half of the safe endurance (*q*). In fact, for each vehicle, the charging process shown in Fig. 21 needs to be performed only every two days. During the redundant charging time and idle time of UFMCs, they can serve as backups for each other. Considering that Ezhou is a specialized cargo airport, the guidance tasks at night at this airport are relatively active, which is related to the characteristics of distribution of freight flight.

## Conclusion

In this study focused on the integrated optimization of scheduling for UFMCs on airport surface, we proposed the concept of GUs and a quantification method for guidance following spacing. We established an integrated UFMCs scheduling model, consisting of trajectory planning and vehicle assignment, considering the demands of precise connecting among guidance tasks and the vehicle charging. Additionally, considering the vehicle dynamics, we designed a high-precision method of guidance trajectory deduction and a conflict prediction model based on protected zones. We also developed an IGWO algorithm fitting the integer encoding.

Secondly, we validated the proposed approaches using the case of Huahu Airport in Ezhou. The IGWO demonstrated significant improvements in generating conflict-free feasible solution sets and generalization performance. The IGWO-1 algorithm showed potential in generating high-quality feasible solution sets for medium-scale optimization of scheduling for UFMCs. 64.44% and 25% of guidance tasks successfully avoided potential conflicts through fine-tuning timing and detour, sacrificing only 2.85% of efficiency to ensure safety. In the vehicle assignment module, the assignment inequality index was as low as 0.015731, indicating the achievement of collaborative planning and the achievement of balanced allocation of guidance tasks.

Finally, future research will explore tactical optimization strategies for flight delays or unexpected incidents encountered by UFMCs, aiming to enhance the robustness of algorithms and models.

## Data Availability

The dataset used and analyzed in the current study is available from the corresponding author on reasonable.
